# Hematopoiesis Remains Permissive to Bone Marrow Transplantation After Expansion of Progenitors and Resumption of Blood Cell Production

**DOI:** 10.3389/fcell.2021.660617

**Published:** 2021-08-03

**Authors:** Martin Báječný, Chia-Ling Chen, Kateřina Faltusová, Tomáš Heizer, Katarína Szikszai, Petr Páral, Luděk Šefc, Emanuel Nečas

**Affiliations:** ^1^1st Faculty of Medicine, Institute of Pathological Physiology, Charles University, Prague, Czechia; ^2^1st Faculty of Medicine, Center for Advanced Preclinical Imaging (CAPI), Charles University, Prague, Czechia

**Keywords:** bone marrow transplantation, stem cell, progenitor cell, regeneration, transferrin receptor, Sca-1 antigen

## Abstract

The immense regenerative power of hematopoietic tissue stems from the activation of the immature stem cells and the progenitor cells. After partial damage, hematopoiesis is reconstituted through a period of intense regeneration when blood cell production originates from erythro-myeloid progenitors in the virtual absence of stem cells. Since the damaged hematopoiesis can also be reconstituted from transplanted hematopoietic cells, we asked whether this also leads to the transient state when activated progenitors initially execute blood cell production. We first showed that the early reconstitution of hematopoiesis from transplanted cells gives rise to extended populations of developmentally advanced but altered progenitor cells, similar to those previously identified in the bone marrow regenerating from endogenous cells. We then identified the cells that give rise to these progenitors after transplantation as LSK CD48^–^ cells. In the submyeloablative irradiated host mice, the transplanted LSK CD48^–^ cells preferably colonized the spleen. Unlike the endogenous hematopoiesis reconstituting cells, the transplanted whole bone marrow cells and sorted LSK CD48^–^ cells had greater potential to differentiate to B-lymphopoiesis. Separate transplantation of the CD150^–^ and CD150^+^ subsets of LSK CD48^–^ cells suggested that CD150^–^ cells had a greater preference to B-lymphopoiesis than CD150^+^ cells. In the intensively regenerating hematopoiesis, the CD71/Sca-1 plot of immature murine hematopoietic cells revealed that the expanded populations of altered myeloid progenitors were highly variable in the different places of hematopoietic tissues. This high variability is likely caused by the heterogeneity of the hematopoiesis supporting stroma. Lastly, we demonstrate that during the period when active hematopoiesis resumes from transplanted cells, the hematopoietic tissues still remain highly permissive for further engraftment of transplanted cells, particularly the stem cells. Thus, these results provide a rationale for the transplantation of the hematopoietic stem cells in successive doses that could be used to boost the transplantation outcome.

## Introduction

The potential to repair damaged tissues is a specific feature of multicellular organisms highly developed during their embryogenesis and maintained to a variable degree to adulthood. In mammals, the regenerative capacity of tissues frequently exposed to mechanical injuries like skin and oral mucosa has been recognized for a long time and, somewhat surprisingly, also for the liver (Power and Rasko, 2008). The high regenerative capacity of bone marrow and intestinal epithelium has been recognized only recently after discovering the tissue-damaging effects of ionizing radiation and cytostatic drugs. The ensuing research resulted in the discovery of developmentally pluripotent hematopoietic stem cells (HSCs) (Ford et al., 1956) and became the basis of bone marrow/hematopoietic cell transplantation, an effective curative treatment for severe diseases (Simpson and Dazzi, 2019). The transplantation of hematopoietic cells precedes a conditioning treatment damaging the hematopoietic tissues of the transplant recipient. In experimental transplantation of hematopoietic cells, the conditioning usually consists of a total body exposure of mice to ionizing radiation. The conditioning can utilize either the myeloablative (lethal) dose of radiation or a submyeloablative dose, allowing for reconstitution of damaged hematopoiesis from endogenous irradiation-surviving cells. The transplantation of hematopoietic cells to recipients conditioned by the submyeloablative damage to hematopoietic tissues results in chimeric hematopoiesis derived from transplanted cells and the hematopoiesis derived from endogenous cells (Forgacova et al., 2013).

We have recently uncovered highly expanded populations of altered hematopoietic progenitor cells in the virtual absence of stem cells in intensively regenerating bone marrow recovering from submyeloablative damage (Faltusová et al., 2020b). These immature hematopoietic cells differ from the progenitor cells present in normal bone marrow by the decreased expression level of the c-Kit receptor for stem cell factor (SCF), by the expression of Sca-1 antigen also in the cells that express transferrin receptor 1 (CD71), by the expression of CD16/32 in most of the LK cells, and by the altered expression of CD41. The progenitors activated the erythroid developmental program independently from erythropoietin production. Despite decreased expression of the c-Kit receptor, the progenitors require effective stimulation by SCF for their expansion. The progenitor cells supported the production of mature blood cells in the virtual absence of transplantable stem cells, which is reminiscent of the first wave of definitive embryonic hematopoiesis proceeding the developmental of the first pluripotent stem cells. At the same time, it is significantly different from the hematopoiesis in the fetal liver supported by stem cells (Faltusová et al., 2020b).

In the experimental model of adult hematopoiesis reconstitution used by Faltusová et al. (2020b), the cells initiating reconstitution were exposed to the direct action of ionizing radiation that could have been harmful (Abramson et al., 1977; Shao et al., 2014). In the present experiments, we have examined the reconstitution of damaged hematopoiesis from transplanted cells that were not exposed to radiation. We asked whether hematopoiesis derived from transplanted hematopoietic cells would also increase populations of altered progenitor cells similar to those that develop in the hematopoiesis regenerating spontaneously from endogenous, radiation-exposed cells. We also attempted to identify the cells giving rise to an expanded progenitor population. Further, we also explored the high inter-mice variability in CD71 and Sca-1 expression in immature hematopoietic cells in regenerating hematopoiesis. To achieve this, we analyzed the chimeric donor/host hematopoiesis. We have also approached whether intensively regenerating hematopoiesis with extended populations of altered progenitor cells would still engraft transplanted hematopoietic cells.

## Materials and Methods

### Mice

C57BL/6J (CD45.2), B6.SJL-PtprcA PepcB/BoyJ (CD45.1), and C57Bl/6-Tg (UBC-GFP) 30Scha/J mice of both sexes were used. The mice were bred in an institutional animal specific-pathogen-free facility and maintained in a clean conventional part of the facility (12:12 h light–dark cycle, 22 ± 1°C, 60 ± 5% humidity) during the experiments. Studies used adult mice (mostly 8–12 weeks old) of both sexes. The experiments were performed by national and international guidelines for laboratory animal care and approved by the Laboratory Animal Care and Use Committee of the First Faculty of Medicine, Charles University and the Ministry of Education, Youth and Sports and of the Czech Republic (MSMT-4502/2017-2).

### Conditioning of Mice for Transplantation by Irradiation

Whole-body irradiation was performed in a plastic cage from a ^60^Co source (Chisobalt 2-B75, Czech Republic) with a dose rate ∼0.35 Gy per minute. The myeloablative irradiation used a dose of 9 Gy. The submyeloablative irradiation, employed to establish chimeric hematopoiesis after transplantation of congenic hematopoietic cells, used doses of 3–7 Gy adjusted to the number of transplanted cells. The particular dose of radiation is indicated in the legends to results.

### Bone Marrow and Spleen Cell Collection

Bone marrow cells were obtained from the long bones (femurs and tibias, or femurs only) by flushing the bone cavity with phosphate-buffered saline (PBS) with 1% bovine serum albumin through a hole in one end of the bone without clipping off the epiphyses. A loosely fitting glass homogenizer was used to release spleen cells into 2 ml of PBS. Cells were filtered across a 70-μm nylon cells filter (BD Biosciences, San Jose, CA, United States). A single-cell suspension was obtained by repeated passage through the needle (25G) and kept at 4°C on ice before further handling.

### Transplantation of Hematopoietic Cells

To minimize cell handling *ex vivo*, whole bone marrow cells to be transplanted were appropriately diluted by PBS shortly after their collection, and a defined fraction of the femoral bone marrow cells was intravenously injected to host mice in a volume of 0.3 ml via the retro-orbital route. Host mice were irradiated 2–4 h before transplantation. When mice were transplanted twice, the syngeneic cells were transplanted 2–4 h after irradiation, and a standard dose of congenic cells corresponding to a half of femur or two femurs was transplanted 2 h–30 days afterward. Transplanted sorted cells were subjected to *ex vivo* handling during their staining and sorting. They were kept at 4°C during the approximately 3-h-lasting procedure.

### Flow Cytometry and Cell Sorting

Cells to be analyzed or sorted were stained with fluorochrome-labeled antibodies in optimal dilutions for 30 min at 4°C in the dark. The antibodies used are listed in Supplementary Table 1. Cells were analyzed or sorted with a FACSAria IIu cell sorter (BD Biosciences) equipped with 488 nm (50 mW), 561 nm (100 mW), 638 nm (140 mW), 405 nm (100 Mw), and 355 nm (20 mW) lasers. BD FACSDiva software version 6.1.3 was used for data acquisition, and debris and dead cells were excluded from the analysis by gating the FSC-A/SSC-A dot plot. For cell doublet discrimination, an FSC-A/FSC-H dot plot was used. Fluorescence-Minus-One (FMO) control samples were used for discrimination of the cells positive for a particular marker. Either a 70- or 85-μm integrated nozzle was used with corresponding sheath pressure for cell sorting. Cells were sorted into polypropylene microcentrifuge tubes (Eppendorf, Hamburg, Germany) containing 1 × PBS supplied with 3–5% albumin fraction V, biotin-free (Carl Roth GmbH, Karlsruhe, Germany). Sterile 1 × PBS was used as the sheath fluid. The flow cytometry data were analyzed by the recent version of FlowJo software (BD Biosciences).

### Analysis of Chimeric Hematopoiesis in Bone Marrow or Spleen

Bone marrow or spleen cells were washed with PBS (centrifuged at 4°C, 400 *g* for 6 min) and stained for 30 min on ice and in the dark with a mixture of fluorescently labeled or biotin-labeled antibodies in optimal dilution. When a biotin-labeled antibody was used, cells were washed and labeled for the second time with streptavidin containing a fluorescent dye. Antibodies labeled with fluorescein isothiocyanate (FITC) were omitted when chimeric hematopoiesis contained green fluorescent protein (GFP)-expressing cells.

### Analysis of Chimeric Hematopoiesis in Peripheral Blood

The ratio of donor to host nucleated blood cells was determined in samples of peripheral blood drawn from the retro-orbital venous plexus into a capillary containing 5 μl of 0.5 M EDTA. Approximately 50 μl of blood was stained with anti-CD45.1 and anti-CD45.2 antibodies for 30 min on ice in the dark and washed after. The samples were also stained for Gr-1/Mac-1, B220, CD4, and CD8 markers. Only CD45.1 or CD45.2 single-positive cells were evaluated by flow cytometry, and double CD45.2/CD45.1-positive cells were excluded from the analysis. When the chimeric blood contained GFP-positive cells and wild-type cells, the FITC-labeled antibodies were omitted. The chimerism in red blood cells and platelets was analyzed only in experiments with GFP-positive and wild-type cells using CD41 and TER119 markers. Blood cells were analyzed using a digital FACS Canto II flow cytometer (BD Biosciences, San Jose, CA, United States), equipped with 405 nm (60 mW), 488 nm (20 mW), and 633 nm (15 mW) lasers.

### Green Fluorescent Protein Fluorescence on Spleen Surface

Pictures of the spleen hematopoiesis derived from transplanted cells of UBC-GFP mice origin were captured by an optical Xtreme *in vivo* imager (Bruker, Ettlingen, Germany) using the 480-nm excitation filter and the 535-nm emission filter. For coloring of the scale of fluorescent intensity, Fiji-ImageJ software was used.

### RT-qPCR Assay

Total RNA was isolated using an RNeasy Plus Mini kit (Qiagen, Hilden, Germany) from cells sorted into PBS. An iScript^*TM*^ cDNA Synthesis kit (BIO-RAD, Hercules, CA, United States) was used for cDNA synthesis. Quantitative PCR was performed using a 7900HT Fast Real-Time Cycler (Applied Biosystems, Foster City, CA, United States) and Biotool^*TM*^ 2xSYBR Green qPCR Master Mix (BioTools, Jupiter, United States). Each sample was done in a triplicate minimally, threshold cycle (CT) values from replicates were calculated as mean CT, and the results were expressed as relative gene expression by 2^–ΔCT^ or 2^–ΔΔCT^ method. Each sample was normalized to the beta-actin (Actb) mRNA level. We used DataAssist Software (Thermo Fisher Scientific, Waltham, MA, United States) and GraphPad Prism to determine and remove the outlier replicate of the CT value. The primers to quantify cDNA levels are indicated in Supplementary Table 2. The gene expression in the samples collected from donor-derived and host-derived cells in the chimeric bone marrow was compared with that in the control samples obtained from the bone marrow of either CD45.2 or CD45.1 untreated mice.

### Statistical Analysis

Statistical analysis was done with GraphPad Prism (GraphPad Software). Two-tailed unpaired Student’s *t*-tests were used for statistical significance when the two groups were compared. Two-way analysis of variance (ANOVA) was used to compare each group to the control group when more than two groups were evaluated. Results are means of all data presented or means ± standard error of the mean (SEM) or standard deviation (SD) as stated in the legends to figures. *P*-values < 0.05 were considered statistically significant. The statistical analysis of gene expression results used an unpaired *t*-test, and the two-tailed statistic calculated *p*-values. The bars show standard deviation (SD). The graphics and the statistics were performed by GraphPad Prism software. The regression analysis of the decline in the permissiveness of regenerating hematopoiesis for engrafting transplanted cells used the log (inhibitor) vs. response curve–variable slope (four parameters) non-linear regression using calculation.

## Results

### Phenotype of LK Cells Derived From Transplanted Bone Marrow Corresponds to Erythro-Myeloid Progenitors in Intensively Regenerating Hematopoiesis

**FIGURE 1:**
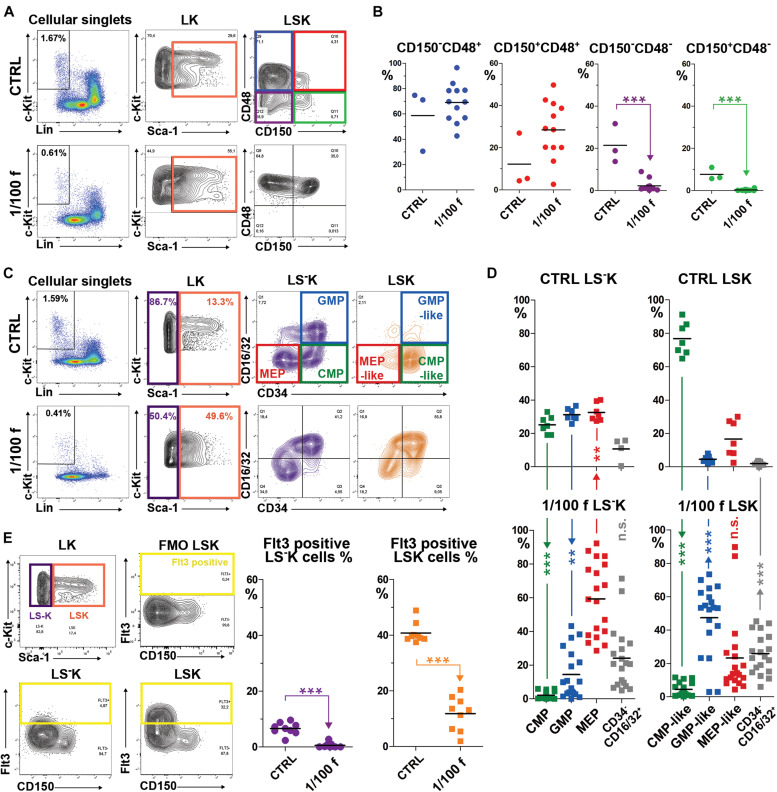
Immature hematopoietic cells in bone marrow regenerating from transplanted cells have significantly altered immunophenotype. **(A)** The gating of LSK cells with four CD150/CD48 expression profiles according to Kiel et al. (2005). **(B)** The four subsets of LSK cells with the specific CD150/CD48 expression profile were analyzed in three independent experiments that each included one untreated mouse (CRTL) and a group of four mice irradiated at 9 Gy, transplanted with 1/100 of the syngeneic femoral bone marrow, and examined after 14 days. **(C)** The gating of the four CD34/CD16-32 expression profiles according to Akashi et al. (2000) in LS^–^K, but also in LSK cells. **(D)** LS^–^K and LSK cells were analyzed for the expression pattern of CD34 and CD16/32 in seven untreated mice (CTRL) and 19 mice transplanted with 1/100 of the syngeneic bone marrow examined after 14 days. Results are pooled from five independent experiments. **(E)** The gating used to determine Flt3 (CD135)-positive cells in LS^–^K and LSK cells in nine untreated mice (CTRL) and nine mice transplanted with 1/100 of the syngeneic femoral bone marrow and examined after 14 days. **p < 0.01, ***p < 0.001.

We asked whether the immature lineage-negative and c-Kit-positive hematopoietic cells (Lin^–^c-Kit^+^; LK) derived from transplanted cells will significantly differ from normal LK cells. Lethally (myeloablative) irradiated mice were transplanted with 1/100 of the femoral bone marrow (≈250,000 cells). Fourteen days after transplantation, bone marrow was collected and examined with the focus on LK cells, which were divided into those expressing Sca-1 antigen (LSK cells) and those lacking Sca-1 (LS^–^K cells) (Figure 1). LSK cells were further characterized by CD150 and CD48 markers into four subtypes (Figures 1A,B). LS^–^K cells were characterized according to the expression of CD34 and CD16/32 markers. These features divide LS^–^K cells into the common myeloid progenitor cells (CD34^+^CD16/32^–^; CMP), progenitor cells committed to megakaryocytic and erythroid development (CD34^–^CD16/32^–^; MEP), and granulocyte/macrophage committed progenitor cells (CD34**^+^**CD16/32^+^; GMP). We analyzed the CD34 and CD16/32 expression in LSK cells unconventionally because Sca-1 is induced in intensively regenerating hematopoiesis. LSK cells were marked as CMP-like, MEP-like, and GMP-like when they showed the CD34/CD16-32 expression pattern corresponding to CMP, MEP, and GMP LS^–^K cells (Figures 1C,D). The percentage of cells positive for CD135 (Flt3) marker was determined in LSK and LS^–^K cells (Figure 1E).

LSK cells virtually lacked CD48^–^ cells (Figure 1B), and a large proportion expressed CD16/32 in regenerating bone marrow (Figure 1D). LSK cells with the immunophenotype of CD34^+^CD16/32**^–^** (CMP-like cells) were significantly suppressed (Figure 1D). Flt3 expression (CD135) in LSK cells dropped from approximately 40% to about 10% in regenerating bone marrow (Figure 1E).

LS^–^K cells with the CMP phenotype were significantly decreased, and those with the phenotype of GMP and MEP increased (Figure 1D) in regenerating bone marrow. Flt3^+^ LS^–^K cells became very rare (Figure 1E).

Collectively, these results revealed the significant alteration of LK cells in the bone marrow intensively regenerating from transplanted cells. The phenotype of LK cells in regenerating bone marrow corresponded mainly to that of GMP and MEP progenitor cells, irrespective if they were Sca-1^–^ or Sca-1^+^.

### CD71/Sca-1 Plot of LK Cells Reveals Activated Myeloid Progenitors in Intensively Regenerating Bone Marrow of Transplanted Mice

**FIGURE 2:**
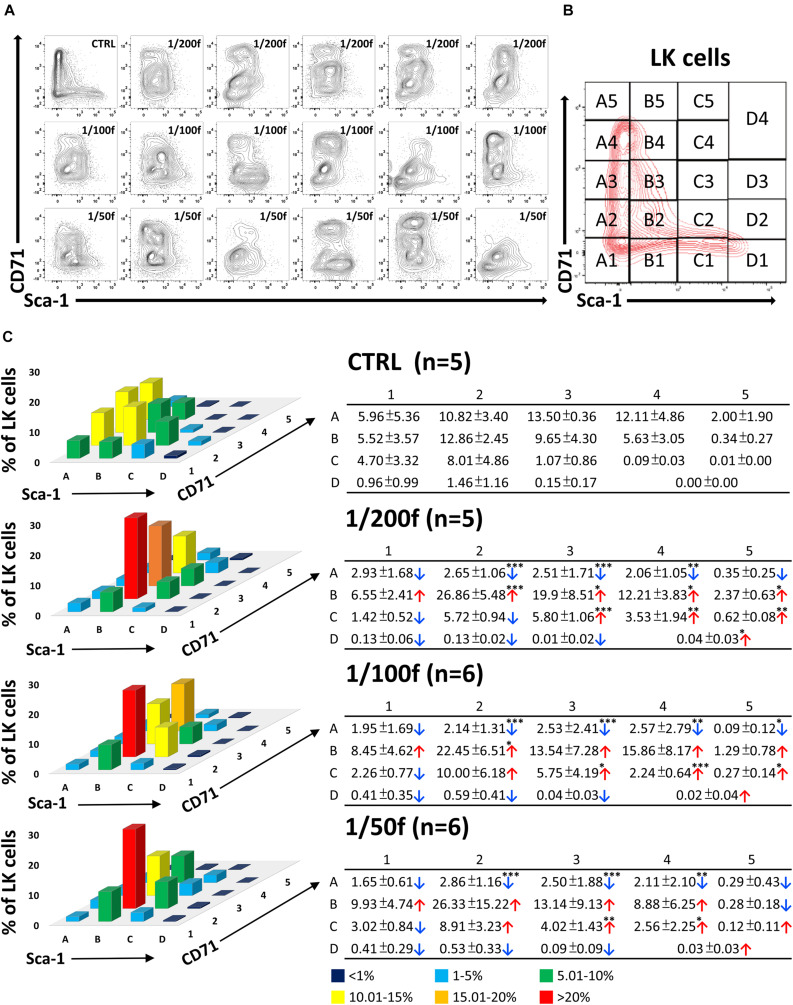
Immature hematopoietic cells derived from transplanted bone marrow have altered immunophenotype in intensively regenerating bone marrow. In two independent experiments, C57Bl/6 female mice irradiated at 9 Gy were transplanted with syngeneic bone marrow corresponding to 1/200 (five mice), 1/100 (six mice), and 1/50 (six mice) of the femoral bone marrow (male). Five untreated mice were left as controls (CTRL; two females and three males). Two weeks after transplantation, LK cells in bone marrow were analyzed to express Sca-1 and CD71 markers **(A)**. The CD71/Sca-1 plot of LK cells was divided into 19 parts **(B)**, and the percentage of LK cells was determined in them. The mean occurrence of cells in various parts of the CD71/Sca-1 plot is graphically shown as the mean values ± SD in accompanying tables **(C)**. Tables show significantly changed cell numbers in some zones of CD71/Sca-1 plot in transplanted mice. ^∗^*p* < 0.05, ^∗∗^*p* < 0.01, ^∗∗∗^*p* < 0.001.

**FIGURE 3:**
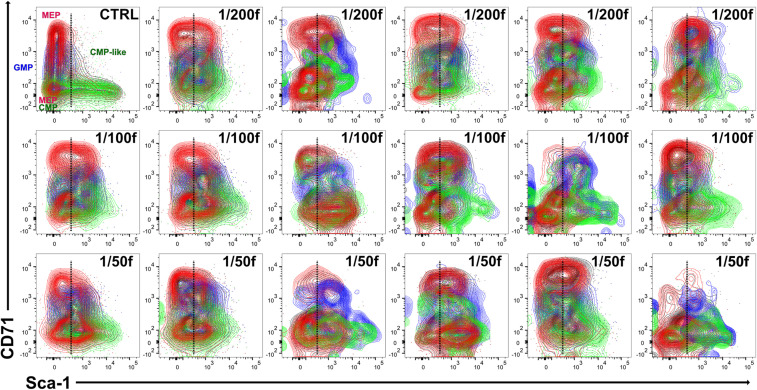
The variable cell clusters of LK cells in regenerating bone marrow have immunophenotype corresponding to CMP, GMP, and MEP progenitors in the normal bone marrow. LK cells in one control mouse (CTRL) and 17 irradiated and transplanted mice (all females) analyzed in Figure 2 were stained for the CD34 and CD16/32 markers correspondingly to CMP (green), GMP (blue), and MEP (red) progenitors known in normal bone marrow.

The CD71/Sca-1 plot of LK cells is highly constant in the normal bone marrow and clearly distinguishes between LSK cells, which are uniformly CD71 negative, and LS^–^K cells with an extensive range of CD71 expression intensity (CTRL samples in Figures 2, 3 and Supplementary Figure 1). We applied the CD71/Sca-1 plot to LK cells in bone marrow regenerating from transplanted cells. This approach revealed the significantly altered and highly variable CD71/Sca-1 profile of LK cells in mice transplanted with 1/200, 1/100, and 1/50 of the femoral bone marrow (Figure 2A). We then analyzed how the distribution of LK cells is altered in 19 groups of LK cells with various CD71 and Sca-1 expression (Figure 2B). Change in the phenotype of LK cells did not depend on the number of transplanted cells, which can be estimated to be ≈125,000–500,000 bone marrow cells (Figure 2C).

We used the CD34/CD16–32 expression profile to localize cells with the phenotype corresponding to CMP, GMP, and MEP (Supplementary Figure 2) in the CD71/Sca-1 plot LK cells (Figure 3). This analysis showed that while in the normal bone marrow, the CD34/CD16-32 phenotype corresponding to GMP and MEP cells is limited to LS^–^K cells; in the regenerating bone marrow, it also occurs in Sca-1-positive (LSK) cells (Figure 3).

Cells with the phenotype of MEP progenitors (red in Figure 3) split into distinct CD71^low^ and CD71^high^ cell clusters. Therefore, we compared the gene expression in the CD71^low^ and CD71^high^ subsets of MEP cells (Supplementary Figure 3). This analysis demonstrated the erythroid developmental commitment in both subsets of MEP cells.

Cells with the phenotype of GMP progenitors were mostly CD71^medium^ cells in both normal and regenerating bone marrow; in the latter, they mainly were Sca-1 positive (Figure 3).

Collectively, these robust results show that bone marrow transplantation gives rise to expanded populations of altered erythro-myeloid progenitors.

### Chimeric Hematopoiesis Reveals Differences in the Reconstitution of Damaged Hematopoiesis Executed by Transplanted or Endogenous Cells

**FIGURE 4:**
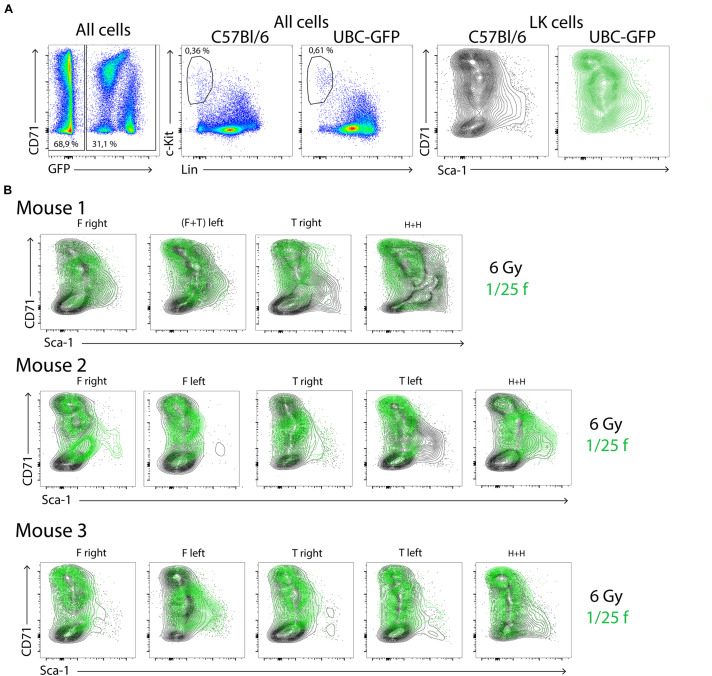
LK cells in chimeric hematopoiesis derived from transplanted and endogenous hematopoiesis reconstituting cells analyzed 2 weeks after irradiation and transplantation. Three C57Bl/6 mice were irradiated at 6 Gy and transplanted with bone marrow of UBC-GFP mice equivalent to 1/25 femur. Bone marrow was collected after 2 weeks separately from the right and left femurs (F), right and left tibias (T), and both humeri (H). **(A)** Aliquots of 15 bone marrow samples were pooled and analyzed. **(B)** LK cells in 15 bone marrow samples of three mice are plotted in the CD71/Sca-1 contour diagram; green, LK cells derived from transplanted (1/25 of the femoral bone marrow) bone marrow of UBC-GFP male mice; black, LK cells derived from endogenous hematopoiesis repopulating cells that survived in the 6-Gy-submyeloablative irradiated hematopoiesis of host C57Bl/6 male mice.

Three C57Bl/6 mice were irradiated at 6 Gy and transplanted with 1/25 of the femoral bone marrow of UBC-GFP mice to compare LK cells derived from transplanted and endogenous hematopoiesis reconstituting cells in various parts of chimeric hematopoiesis. Bone marrow was collected separately from femurs, tibias, and humeri after 2 weeks and processed for analysis by flow cytometry. Figure 4A shows GFP^+^ and GFP^–^ cells in the pooled bone marrow from 15 bone marrow samples obtained from a total of three mice. There were 31.1% of GFP^+^ cells and 68.9% of GFP^–^ cells in this pooled bone marrow sample. GFP^+^ LK cells formed 0.61% of all GFP^+^ cells, and GFP^–^ LK cells formed 0.36% of all GFP^–^ cells. The major difference between LK cells derived from transplanted and endogenous hematopoiesis reconstituting cells was the lack of CD71^low/–^Sca-1^low^ cells in the LK cells derived from transplanted cells. An overlay of CD71/Sca-1 plots of LK cells derived from transplanted (green) and endogenous repopulating cells (black) in 15 samples of bone marrow reveals their high variability between sites of the bone marrow and individual mice (Figure 4B).

In the next experiment, we compared LK cells derived from transplanted cells of UBC-GFP mice and endogenous hematopoiesis repopulating cells of wild-type hosts, also in the spleen. The CD71/Sca-1 profile of LK cells in the spleen differed significantly from that in the regenerating bone marrow and was similar to the profile in normal bone marrow (Supplementary Figure 4).

### Transplanted LSK CD48^–^ Cells Give Rise to Clusters of Erythro-Myeloid Progenitors

To learn which type of LK cells reconstitute hematopoiesis after transplantation after 2 weeks, we sorted LS^–^K cells, LSK CD48^+^, and LSK CD48^–^ cells from the bone marrow of UBC-GFP mice and transplanted them to C57Bl/6 wild-type mice irradiated at 6 Gy. Only LSK CD48^–^ cells significantly competed with endogenous cells in the reconstitution of hematopoiesis in the bone marrow and spleen (Supplementary Figure 5).

Transplanted LSK CD48^–^ cells produced significant numbers of mature red blood cells, GM cells, and platelets 2 weeks after transplantation (Table 1). Of note, 50,000 of LS^–^K cells gave rise to a significant number of red blood cells (19.3% of Ter119^+^ cells in the peripheral blood were GFP^+^), and 8.7% of nucleated cells were GFP^+^ in the spleen and 2.0% in the bone marrow but did not generate LK cells (results from a single mouse).

**TABLE 1:** The percentage of GFP^+^ red blood cells, granulocyte–macrophages, and platelets in the peripheral blood 2 weeks after transplantation of 400 LSK CD48^–^ cells.

	Ter119	GM	CD41
PB	**8.82** ± 1.81%	**4.92** ± 5.42%	**11.91** ± 4.79%
BM	**7.02** ± 4.47%	**2.01** ± 0.76%	**2.77** ± 0.86%
Spleen	**48.8** ± 2.12%	**30.3** ± 2.73%	**9.71** ± 1.27%

*PB, peripheral blood; BM, bone marrow. The values are mean (bold) ± SD.*

**FIGURE 5:**
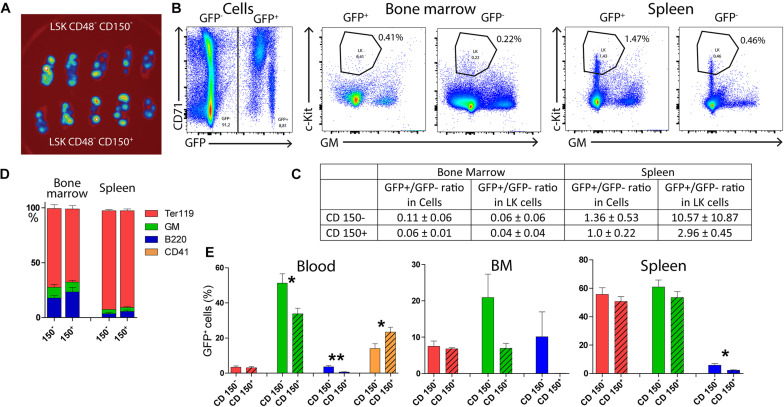
Both CD150^+^ and CD150^–^ LSK CD48^–^ cells reconstitute damaged hematopoiesis. Ten female C57Bl/6 mice were irradiated at 6.5 Gy and transplanted with 400 LSK CD48^–^, either CD150^–^ or CD150^+^ cells sorted from the bone marrow two UBC-GFP mice. Two weeks after transplantation, the peripheral blood and hematopoietic tissues were collected and analyzed. **(A)** Spleens visualized for green fluorescent protein (GFP) fluorescence. **(B)** Examples of the gating of LK cells in the chimeric bone marrow and spleen cells. **(C)** Ratio between GFP^+^ and GFP**^–^** cells in all cells and LK cells. **(D)** The proportion of all (GFP^+^ and GFP^–^ cells, not distinguished) Ter119^+^ (erythroblasts), GM^+^ (myelocytes and monocytes), and B220^+^ (B cells) cells in the bone marrow and spleen of transplanted mice. **(E)** The occurrence of GFP^+^ red blood cells (Ter119), granulocytes–macrophages (GMPs), B lymphocytes (B220), and platelets (CD41) in the peripheral blood. *p < 0.05, **p < 0.01. See Supplementary Figures 6, 8 for the gating used for sorting cells for transplantation and analysis of red blood cells and platelets in the blood.

Furthermore, we compared the capacity of CD150^+^ and CD150^–^ subsets of LSK CD48^–^ cells to reconstitute submyeloablative damaged hematopoiesis. Ten female C57Bl/6 mice were irradiated at 6.5 Gy and transplanted with 400 LSK CD48^–^ either CD150^–^ (five mice) or CD150^+^ (five mice) cells sorted from the bone marrow of two UBC-GFP female untreated donors. The gating used for the cell sorting is shown in Supplementary Figure 6. After 14 days, a blood sample was obtained from the retro-orbital venous plexus, bone marrow was collected from both femurs, and spleens. The spleens were examined for GFP fluorescence before releasing their cells. The peripheral blood, bone marrow cells, and spleen cells were analyzed to identify GFP-positive cells. Results are shown in Figure 5. Supplementary Figure 7 then shows the overlay of GFP^+^ and GFP^–^ LK cells in the CD71/Sca-1 diagram, and Supplementary Figure 8 shows how GFP^+^ and GFP^–^ red blood cells and platelets were distinguished in the blood.

This experiment showed that transplanted LSK CD48^–^ cells engraft preferentially in the spleen irrespective of the CD150^–^ or CD150^+^ phenotype, while the bone marrow hematopoiesis is reconstituted primarily from endogenous repopulating cells of the host. There was no apparent difference in the hematopoiesis reconstituting capacity of CD150^–^ or CD150^+^ LSK CD48^–^ cells 2 weeks after transplantation, and both gave rise to irregular clusters of CD71/Sca-1 LK cells in the bone marrow.

### CD150^–^ LSK CD48^–^ Cells Are Lymphoid-Biased as Compared With CD150^+^ LSK CD48^–^

There were more GFP^+^ B cells in the peripheral blood after transplantation of LSK CD48^–^ CD150^–^ cells than the transplantation of their CD150^+^ counterparts (Figure 5). We also compared the outcome of transplantation of CD150^+^ and CD150^–^ LSK CD48^–^ cells sorted from the bone marrow of CD45.2 female mice and transplanted to irradiated (7 Gy) CD45.1 female mice. The resulting chimeric CD45.2/CD45.1 hematopoiesis was evaluated after 3 weeks.

In mice transplanted with CD150^+^ cells, 96.7 ± 2.2% of GM cells and 86.7 ± 9.0% of B220 cells were donor origin (CD45.2) in the bone marrow. In mice transplanted with CD150^–^ cells, 71.6 ± 23.3% of GM cells and 89.3 ± 5.1% of B220 cells were of donor origin. The GM/B220 ratio in bone marrow cells was 5.20 ± 2.00 after transplantation of CD150^+^ cells and 1.43 ± 0.36 after transplantation of CD150^–^ cells.

In the peripheral blood, donor CD45.2 cells comprised 89.8 ± 5.5% of GM cells but only 7.8 ± 5.4% of B220 cells after transplantation of CD150^+^ cells. After transplantation of CD150^–^ cells, 47.0 ± 1.3% of GM cells and 50.2 ± 0.9% of B220 cells were of donor origin in the blood (Supplementary Figure 9).

LK cells were more abundant after transplantation of CD150^+^ cells (0.83 ± 0.19%) than after the transplantation of CD150^–^ cells (0.44 ± 0.29%) in the bone marrow. The difference was also in the proportion of LSK CD150^+^CD48^–^ cells in all LSK cells. It was 16.30 ± 3.48% after the CD150^+^ cell transplantation and 5.55 ± 0.71% after the CD150^–^ cell transplantation. These results are also shown in Supplementary Figure 9. These experiments confirm that both LSK CD48^–^CD150^+^/CD150^–^ cells can acutely reconstitute hematopoiesis after transplantation, but the developmental potential of CD150^–^ cells is biased for lymphopoiesis compared with CD150^+^ cells.

### Gene Expression Suggests Differences Between LK Cells Derived From Transplanted and Endogenous Cells

Chimeric hematopoiesis was established in CD45.1 submyeloablatively irradiated female mice by transplantation of bone marrow of CD45.2 male donors. Three weeks after transplantation, RNA was obtained from sorted LSK and LS^–^K cells separately from CD45.2 and CD45.1 bone marrow cells. In parallel, RNA was obtained from LSK and LS^–^K cells sorted from the bone marrow of CD45.1 and CD45.2 age- and sex-matched untreated mice as control samples to the samples from the chimeric bone marrow. RNA was processed for the analysis of mRNA levels of selected genes related to undifferentiated cells and cells committed to GMP, lymphocyte (Lym), megakaryocyte-erythroid (MEP), and erythroid (EP) developmental lineages.

**FIGURE 6:**
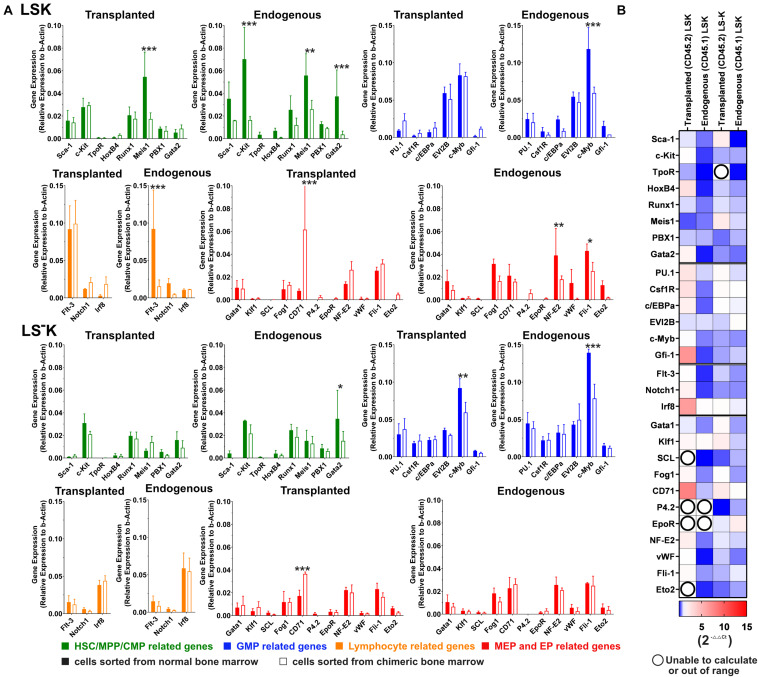
Expression of selected genes in LSK and LS^–^K cells in chimeric hematopoiesis derive from transplanted and endogenous cells. **(A)** A 1/12 of femoral bone marrow (CD45.2) was transplanted to 6-Gy-irradiated CD45.1 mice (both females). The aim was to achieve an equal number of both cell types. There were approximately 65% of CD45.2 cells in the resulting chimeric bone marrow. The chimeric bone marrow was collected after 3 weeks, and CD45.1 and CD45.2 LSK and LS^–^K cells were sorted and their RNA analyzed by qR-PCR (2^–ΔCT^). Solid column, control samples from LSK and LS^–^K cells of untreated mice; empty columns, LSK and LS^–^K cells from chimeric hematopoiesis, derived from transplanted or endogenous hematopoiesis reconstituting cells. The CD45.1/CD45.2 chimeric bone marrow was collected and pooled from four mice. The control CD45.1 bone marrow was pooled from three mice, and the CD45.2 control bone marrow was pooled from two mice. The bone marrow cells were collected from both tibias and femurs. Data represent the average of three to eight duplicates. Error bars represent SD. **(B)** Gene expression heatmap (2^–ΔΔCT^): red, increased gene expression; blue, decreased gene expression; numbers show the fold change compared with control samples; white = 1 indicates no difference in gene expression between the experimental and the control samples; empty circles indicate results in which one of the compared values was out of the scale. GraphPad Prism created the heatmap. *p < 0.05, **p < 0.01, ***p < 0.001.

The results in Figure 6 compare the gene expression level between control (full columns) and chimeric regenerating bone marrow (empty columns) in LSK and LS^–^K cells. The results are also presented in the form of a heatmap (Figure 6B). The heatmap suggests a different gene activity in LSK cells derived from transplanted and endogenous cells in this limited set of examined genes.

### LSK CD150^–^CD48^–^ Cells Are Suppressed in the Hematopoiesis Reconstituted From Transplanted or Endogenous Host Cells

**FIGURE 7:**
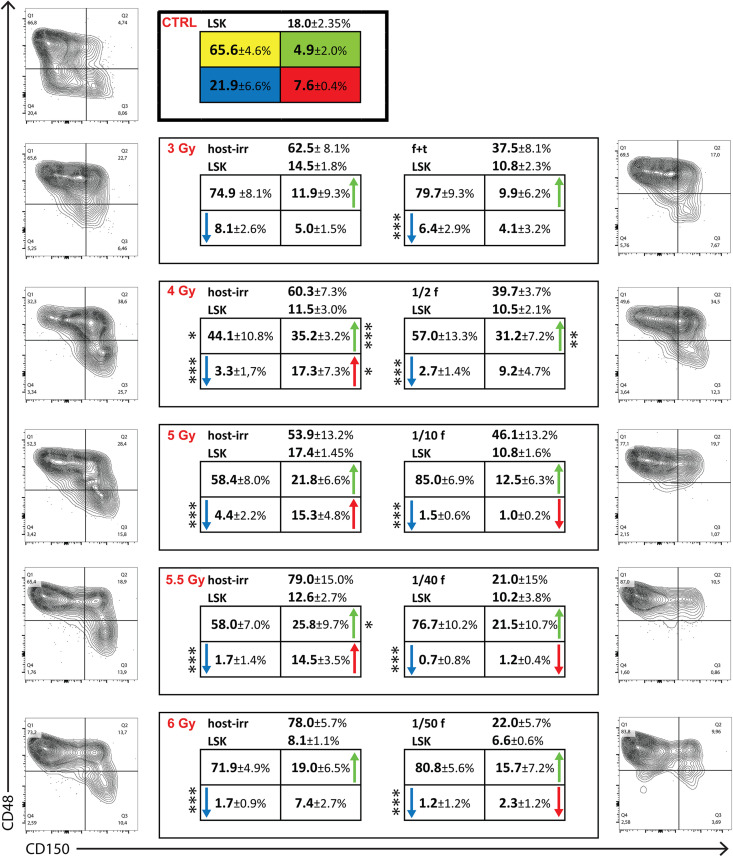
LK cells in chimeric bone marrow reconstituted from transplanted and endogenous cells 1 month after transplantation. Groups of 3 CD45.2 male mice were irradiated at 3, 4, 5, 5.5, or 6 Gy and transplanted with bone marrow of CD45.1 male mice in an amount equivalent to that in femur and tibia, 1/2 femur, 1/10 femur, 1/40 femur, and 1/50 femur. Bone marrow was collected after 1 month, stained for CD45.2, CD45.1, differentiation lineages, c-Kit, Sca-1, CD150, and CD48 markers. The percentage of CD45.2 (host-irr) and CD45.1 (transpl) is shown in LK cells and the percentage of Sca-1-positive (LSK) cells. LSK cells are divided into four CD150/CD48 subtypes, and the percentage of each is shown as mean ± SD. Representative CD150/CD48 plots of LSK cells are shown. CTRL is for three untreated mice (CD45.2); host-irr is for the hematopoiesis derived from endogenous cells in submyeloablatively irradiated (CD45.2) mice; tanspl is for the hematopoiesis derived from transplanted (CD45.1) bone marrow cells. *p < 0.05, ***p < 0.001.

In the next experiment, we determined the reconstitution of four CD150/CD48 subsets of LSK cells in chimeric donor/host hematopoiesis 1 month after conditioning the hosts by irradiation and transplantation of congenic bone marrow cells. Groups of three CD45.2 mice were irradiated with 3, 4, 5, 5.5, or 6 Gy and transplanted with progressively decreasing amount of bone marrow of CD45.1 mice, that of one femur and tibia, 1/2 femur, 1/10 femur, 1/40 femur, and 1/50 femur in the attempt to establish a 50:50 donor/host chimeric hematopoiesis. After 1 month, LK cells were analyzed separately in CD45.2 and CD45.1 bone marrow cells (Figure 7). The constant finding was the significant deficit in LSK CD150^–^CD48^–^ cells. The decreased proportion of LSK CD150^–^CD48^–^ cells persists in the reconstituted hematopoiesis for long time and also occurs in the hematopoiesis in untreated old mice (our unpublished results). The results shown in Figure 7 might thus reflect an accelerating aging of reconstituted hematopoiesis. LSK CD150^+^CD48^–^ cells recovered from endogenous host cells faster than from donor cells. These results suggest delayed reconstitution of the population of LK cells from transplanted cells compared with their host counterparts and significantly delayed reconstitution of CD150^–^CD48^–^ LSK cells.

### Hematopoiesis Conditioned by Irradiation Allows for Repeated Bone Marrow Transplantation

**FIGURE 8:**
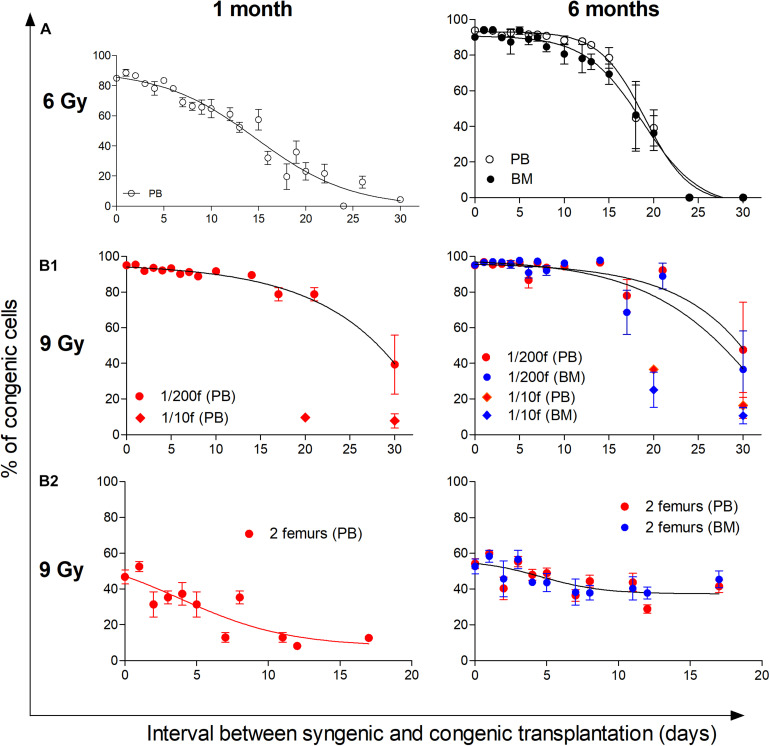
Hematopoiesis remains permissive to transplantation during its intensive regeneration and resumption of blood cell production. The top part of the figure **(A)** shows the previously published results, which determined the decline in the permissiveness to engraftment of transplanted cells in 6-Gy-irradiated mice reconstituting hematopoiesis from endogenous cells (Faltusová et al., 2018). The bottom part of the figure **(B1,B2)** shows results from 9-Gy-irradiated mice receiving double transplants. The first transplantation used syngeneic bone marrow cells delivered shortly after irradiation. The second transplant consisted of congenic bone marrow delivered after 2 h and up to 30 days after the transplantation of syngeneic cells. **(B1)** Irradiated recipient mice were CD45.2, and the syngeneic cells were transplanted in an amount corresponding to 1/200 or 1/10 of the femoral marrow. Congenic bone marrow was transplanted in the amount corresponding to a half of femur. Results are pooled from four independent experiments. **(B2)** Irradiated recipient mice (CD45.1) were transplanted with syngeneic bone marrow from two femurs. The congenic bone marrow collected from CD45.2 mice was delivered in the same amount of two femurs after 2 h and various times till 17 days. Results are from three independent experiments. In both experiments **(B1,B2)**, a sample of the peripheral blood (PB) was collected 1 month after the transplantation of congenic cells, and the percentage of congenic nucleated cells primarily indicated the engraftment of progenitors. The percentage of congenic cells was again determined in the peripheral blood and bone marrow (BM) 6 months after the transplantation of congenic bone marrow cells and indicated the engraftment of hematopoietic stem cells. Data show means ± SEM (*n* = 5–8).

We have previously determined how the permissiveness to bone marrow transplantation is reduced during the spontaneous hematopoiesis regeneration in 6- and 4-Gy-irradiated mice (Faltusová et al., 2018). In the present experiments, we extended this research to the hematopoiesis regeneration derived from transplanted bone marrow. In the initial experiments, myeloablative irradiated mice (9 Gy) were rescued by transplantation of syngeneic bone marrow cells (1/200 or 1/10 of the femoral bone marrow). At various times after this “rescue” transplantation, from 2 h till 30 days, the second transplant of congenic bone marrow cells (half of the femur; 100-fold or 5-fold larger) was given to these mice, and after 1 and 6 months, the congenic blood cells were determined in the peripheral blood, and after 6 months also in the bone marrow (Figure 8B1). The hematopoietic tissues were still highly permissive for engraftment of another bone marrow transplant 2 weeks after transplantation of syngeneic bone marrow, i.e., at the time when bone marrow already contained highly expanded populations of altered erythro-myeloid progenitors (see Figures 1–3).

We tested whether an initial 400-fold higher number of transplanted syngeneic bone marrow cells (two femurs) will rapidly reduce the effectiveness of the second transplant of congenic bone marrow also delivered in the amount of two femurs. The results in Figure 8B2 demonstrate that only the engraftment determined 1 month after transplantation in the peripheral blood, reflecting the hematopoietic activity of transplanted progenitors (short-term repopulating cells), was significantly reduced. On the other hand, the permissiveness for engraftment of stem cells (long-term repopulating cells), reflected by congenic cells in the blood and bone marrow after 6 months, remained virtually unchanged for 17 days after transplantation of syngeneic bone marrow.

These results demonstrate that the permissiveness of hematopoiesis for engraftment of transplanted hematopoietic cells persists in mice conditioned by irradiation when hematopoiesis established expanded populations of altered erythro-myeloid progenitors and became highly active in the production of mature blood cells. In contrast, transplanted stem cells do not significantly expand during this period of hematopoiesis regeneration, allowing for their continued selective engraftment.

## Discussion

The adult hematopoiesis regenerating from a small number of founder cells resembles embryonic and fetal hematopoiesis by the concurrent expansion of immature cells, requiring their self-renewal, and the expansion of blood cell production, requiring differentiation of the immature cells. This specific state of hematopoiesis was why we previously studied the reconstitution of hematopoiesis in mice exposed to the high dose of ionizing radiation, however, still enabling the spontaneous hematopoiesis reconstitution from a tiny number of cells that survived in hematopoietic tissues (Faltusová et al., 2020b). In the present study, we have extended this research to the reconstitution of hematopoiesis from a small number of transplanted cells.

The study’s main goal was to reveal whether transplanted hematopoietic cells give rise to expanded populations of myeloid progenitors during the intensive reconstitution of damaged hematopoiesis. We also tried to understand why the clusters of progenitor cells revealed in the CD71/Sca-1 plot of immature hematopoietic cells in regenerating bone marrow are variable. Finally, we determined how the reconstitution of damaged hematopoiesis interferes with its permissiveness for the engraftment of transplanted cells.

Transplanted bone marrow cells gave rise to altered progenitor cells in intensively regenerating bone marrow similar to the progenitors derived from endogenous hematopoiesis reconstituting cells that survived after exposure to ionizing radiation. The progenitors had decreased expression level of c-Kit and converging Sca-1-positive and Sca-1-negative cells; LSK cells that expressed CD71 and CD16/32 and lacked CD48 had decreased expression of CD135 (Flt3). The phenotypes of the immature lineage-negative and c-Kit-positive cells (LK cells) corresponded mainly to GMP and MEP cells in the normal bone marrow. The regenerating adult hematopoiesis has in common some features/characteristics for the transient embryonic hematopoiesis when EMPs produce blood cells in the absence of stem cells (McGrath et al., 2015; Palis, 2016). Though the LK cells in regenerating bone marrow are distinct from those in the normal bone marrow, they are also distinct from EMPs originating in the yolk sac (McGrath et al., 2015) by not being uniformly CD41^+^, Sca-1^–^, and CD150^–^.

A large part of LK cells in regenerating bone marrow expresses CD16/32, a marker of Sca-1-negative GMPs in the normal bone marrow (Akashi et al., 2000). We sorted CD16/32^+^ LK cells, either Sca-1^–^ or Sca-1^+^, from bone marrow collected from mice that had been irradiated at 6 Gy before 13 days. The *in vitro* colony-forming potential of the cells was compared with CMPs, MEPs, and GMPs (all Sca-1 negative) sorted from the bone marrow of untreated mice. The potential of CD16/32^+^ LK cells from regenerating bone marrow to generate colonies of hematopoietic cells was similar to that of normal GMPs. However, the Sca-1^+^ CD16/32^+^ LK cells from the regenerating bone marrow gave rise to some erythroid colonies and large mixed colonies (Supplementary Figure 10).

The finding that LSK cells express CD71 (transferrin receptor 1; Tfr1) in regenerating bone marrow is best understood in the recent demonstration that Tfr1 is essential for the regeneration potential of HSCs (Wang et al., 2020). The increased expression of Sca-1 in bone marrow has been reported in several studies. Simonnet et al. (2009) demonstrated an increased Sca-1 expression in immature side population (SP) cells in submyeloablatively irradiated mice. Increased Sca-1 expression level in Lin^–^c-Kit^+^CD150^+^ bone marrow cells stimulated by INFα was reported by Essers et al. (2009). Vainieri et al. (2016) demonstrated a massive occurrence of Sca-1^+^ cells in the bone marrow of mice affected by malaria. Notably, these authors attempted to resolve whether the increased proportion of Sca-1^+^ cells resulted from the population expansion of *a priori* Sca-1^+^ cells or the induction of Sca-1 expression in originally Sca-1-negative cells. They concluded that both mechanisms were involved. Walasek et al. (2013) exposed Sca-1-negative CMP, GMP, and MEP cells to three kinds of histone deacetylase inhibitors for 24 h and demonstrated Sca-1 re-induction in CMP and GMP progenitors. Notably, the CMP and GMP cells that re-acquired Sca-1 expression possessed increased self-renewal capacity. The Sca-1 induction in the CD71-positive LK cells in regenerating hematopoiesis thus indicates their activation and possibly increased self-renewal.

The CD71/Sca-1 plot revealed irregular cell clusters in regenerating bone marrow, highly variable between mice and various parts of the bone marrow. The occurrence of cell clusters was a robust phenomenon. They occurred regularly in the regenerating bone marrow in mice of both sexes and were similar when derived from endogenous or transplanted cells, and their occurrence was not significantly affected by the number of transplanted cells. They were generated equally by the transplantation of whole bone marrow cells, LSK CD48^–^ cells, and their CD150^–^ and CD150^+^ subsets. In the spleen, the CD71/Sca-1 diagram differed from that in the bone marrow and was less variable. From all this evidence, we speculate that the primary determinant of the cell type forming these CD71/Sca-1 cell clusters of progenitor cells is heterogeneity in the stromal support provided to regenerating hematopoiesis. The ionizing radiation also damages the stroma of hematopoietic tissues, and it undergoes reconstitution in parallel with the reconstitution of the proper hematopoiesis (Li et al., 2008; Dominici et al., 2009; Hooper et al., 2009; Green et al., 2012). If this hypothesis were correct, the results in Supplementary Figure 1 would suggest that the major phase of stroma reconstitution after submyeloablative irradiation lasts in mice for approximately 20 days.

The occurrence of the variable clusters of progenitor cells may reflect the expanded stress erythroid progenitors characterized by Harandi et al. (2010). The cluster heterogeneity and the requirement for the production of other types of blood cell next to red blood cells suggests that an analogy to the stress erythropoiesis (Paulson et al., 2020) may exist in the other lineages of myeloid blood cells. We had extensively used a model of chimeric hematopoiesis when hematopoiesis was reconstituted in parallel from transplanted cells, not irradiated but manipulated *ex vivo*, and endogenous cells, exposed to radiation but remaining all the time in their tissue context. The ionizing irradiation damages cells by introducing double-strand breaks in DNA and by damaging other cellular components by generated free radicals. This damage causes apoptosis in severely damaged cells (Christophorou et al., 2006) and senescence in stem cells that survive the radiation exposure (Shao et al., 2014). Some HSCs carry chromosomal aberrations after exposure of mice to ionizing radiation but maintain the capacity to reconstitute several lineages of blood cells after transplantation (Abramson et al., 1977). On the other hand, transplanted cells are subjected to *ex vivo* conditions, including oxygen stress, which can induce the differentiation of stem cells (Mantel et al., 2015).

To establish chimeric hematopoiesis, we used two experimental models. In the first, whole bone marrow cells or sorted cells from transgenic UBC-GFP mice were transplanted to wild-type mice. In the second, the donors of hematopoietic cells for transplantation and irradiated host mice had the CD45.2 or CD45.1 allotypes of the CD45 marker. The advantage of the first model is in the very distinct discrimination of cells derived from transplanted cells from those derived from endogenous cells throughout the whole spectrum of hematopoietic and blood cells. The eGFP transgene is located in the MHC locus on chromosome 17 in UBC-GFP mice (Liu et al., 2020), and it appears to negatively affect the stem cells committed to lymphopoiesis, particularly the T-lymphopoiesis (Faltusová et al., 2018, 2020a). The second model has been widely used in experimental hematology research. Its limitation is in the partial immunogenicity of CD45.2 and CD45.1 proteins (van Os et al., 2001; Forgacova et al., 2013; Mercier et al., 2016), reduced reconstituting potential in B cells of CD45.1 origin (Jafri et al., 2017), and the lack of CD45 expression in red blood cells and platelets. CD45 was also less expressed in LK cells in intensively regenerating hematopoiesis 2 weeks after its damage by our experience. Therefore, we used the CD45.2/CD5.1 model to analyze chimeric hematopoiesis only after 3 weeks and 1 month after transplantation. We used mice of both sexes interchangeably in independent experiments to verify the robustness of the results.

By transplanting sorted subtypes of immature hematopoietic cells, we tried to identify the cells that give rise to CD71/Sca-1 clusters of progenitor cells in intensively regenerating hematopoiesis 14 days after transplantation. LSK CD48^–^ cells were the most potent cells in this respect, and both their CD150^–^ and CD150^+^ subsets were similarly effective. The chimeric hematopoiesis established by the transplantation of LSK CD48^–^ cells to partially damaged hematopoiesis was significantly asymmetric in the bone marrow and spleen. The bone marrow utilized for reconstitution of hematopoiesis more endogenous repopulating cells, while transplanted LSK CD48^–^ cells induced hematopoiesis mainly in the spleen. The activation of the splenic milieu for the support of hematopoiesis derived from transplanted cells was first demonstrated in the classical work of Till and McCulloch (1961). The spleen’s preference of transplanted cells to the extramedullary milieu was demonstrated by Andrade et al. (2011). Singbrant et al. (2020) have recently demonstrated that spleen damaged by ionizing radiation strongly activates early erythroid progenitors BFU-E in a bone morphogenic protein 4-responsive manner.

The transplantation of CD150^–^ and CD150^+^ LSK CD48^–^ cells of UBC-GFP mice origin to wild-type mice suggested a mild lymphoid developmental bias of CD150^–^ cells and megakaryocytic bias of CD150^+^. The lymphoid bias of CD150^–^ LSK CD48^–^ cells was also supported in the experiment that used the CD45.2/CD45.1 experimental model of chimeric hematopoiesis, and the hematopoiesis was examined 3 weeks after transplantation. As this was not a primary goal of the study, these findings have not been further elaborated. Weksberg et al. (2008) published similar results 4 and 8 weeks after transplantation of either CD150^–^ or CD150^+^ cells.

Expression of the selected genes that had been studied in our previous research in the LSK and LS^–^K cells in bone marrow 2 weeks after mouse irradiation (Faltusová et al., 2020b) was here studied 3 weeks after irradiation and transplantation in the LSK, and LS^–^K cells were sorted from chimeric hematopoiesis. The differences in gene expression against LSK and LS^–^K cells from normal bone marrow were less pronounced. Also the differences between the gene expression in LSK and LS^–^K cells derived from the transplanted and the endogenous hematopoiesis repopulating cells were small. The LSK cells arising from transplanted cells had normal expression of the genes related to lymphopoiesis. In the LSK cells arising from endogenous cells, the genes were inhibited. The constant finding in the previous study (Faltusová et al., 2020b) and the present study is the upregulation of the gene Gfi-1 in LSK cells in regenerating bone marrow.

The gene expression in LSK and LS^–^K cells from chimeric regenerating bone marrow was compared with the gene expression in LSK and LS^–^K cells from normal CD45.2 or CD45.1 mice (Figure 6). The heatmap (Figure 6B) suggests a different response of some genes in the transplant-derived cells and endogenous cells. However, most of these differences did not reach statistical significance from the particular gene expression in LSK and LS^–^K cells from normal CD45.2 and CD45.1 mice. When these results are compared with our previous results from bone marrow regenerating in mice irradiated at 6 Gy before 2 weeks (Faltusová et al., 2020b), the “erythroid genes” are less upregulated in the present results, which refer to the bone marrow analyzed after 3 weeks following irradiation and transplantation.

Whole-body irradiation by myeloablative and submyeloablative doses of radiation is highly effective in making hematopoiesis permissive to engraftment of transplanted hematopoietic cells (Forgacova et al., 2013). We have determined how the transplantation permissiveness is influenced by the progressive reconstitution of hematopoiesis and demonstrate that the increased permissiveness for engraftment of transplanted cells extends to the period when intensive hematopoiesis is resumed.

The hematopoiesis reconstitution from transplanted cells was delayed compared with the reconstitution from endogenous cells. This delay manifested as a lower proportion of LSK CD48^–^CD150^+^ cells in bone marrow (see Figure 7) and the prolonged permissiveness of the hematopoiesis reconstitute from transplanted cells to accept secondary transplanted cells (Figure 8B1). There are at least three possibilities for this difference. The first possibility is that the number of cells initiating the reconstitution was higher in the case of endogenous cells. We tried to balance the number of transplanted hematopoiesis-reconstituting cells to the endogenous cells by following considerations. Total body irradiation at 6 Gy was lethal to approximately 5% of mice in the first 3 weeks in our previous experiments (Faltusová et al., 2020b). McCarthy (1997) demonstrated that irradiation of mice at 6 Gy induced monoclonal hematopoiesis derived from one stem cell in some mice. Consequently, we estimated that approximately 10–20 stem cells survived in 6-Gy-irradiated mice. Brecher et al. (1993) demonstrated that 10,000–20,000 bone marrow cells contained mostly zero to one stem cell. Therefore, we considered the transplantation dose of 1/200 of syngeneic femoral bone marrow (approximately 125,000 cells) equivalent to endogenous hematopoiesis-reconstituting cells in 6 Gy irradiated mice. However, approximately 20% of myeloablative (lethally) irradiated mice transplanted with 1/200 femoral bone marrow died during the first 2 weeks. Therefore, in further experiments, the dose of transplanted bone marrow cells was increased to 1/100, 1/50, and 1/25 of femoral bone marrow. The second possibility for the delayed reconstitution of hematopoiesis from transplanted cells is that different cells initiated the reconstitution. The third possibility is that while endogenous cells initiated the reconstitution in the bone marrow from the very beginning (Peslak et al., 2012; Faltusová et al., 2020b), the transplanted cells first expanded in the spleen before colonizing the rest of the hematopoietic tissues (Figure 5; Harandi et al., 2010; Andrade et al., 2011).

Of particular interest is the finding that the pool of HSCs do not expand for a long time after transplantation. Mechanistically viewed, transplanted HSCs do not expand and spread to other stem cell niches for a long time after transplantation. A significant number of stem cell niches thus remain available for other transplanted HSCs. In contrast, the pool of progenitors progressively expands in the first 2 weeks after transplantation, which leads to reduced engraftment of transplanted progenitors (Figure 8B2). Thus, this finding is essential for stem cell-based therapy because it shows that the highly active hematopoiesis can engraft transplanted HSCs selectively.

**FIGURE 9:**
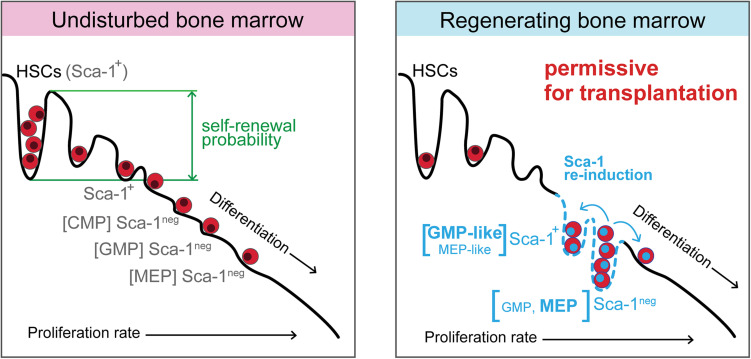
Model of regenerating hematopoiesis driven by activated progenitor cells, which maintain hematopoiesis permissive for transplantation of hematopoietic cells. In the intensively regenerating hematopoiesis that has already resumed blood cell production, the expanded activated progenitor cells diminish the differentiation pressure on multipotent progenitors and stem cells. A similarity can be found in such rearranged hierarchy of immature hematopoietic cells with the period of embryonic hematopoiesis when erythro-myeloid progenitors produce blood cells in the absence of stem cells. The hematopoiesis still remains permissive for the engraftment of transplanted cells, although it became the significant source of mature blood cells.

Collectively, our present and previous (Faltusová et al., 2020b) studies demonstrate that the reconstitution of damaged adult hematopoiesis includes a period when hematopoiesis is supported by activated progenitor cells (Figure 9). This rearrangement of the immature hematopoietic cells hierarchy likely enables less differentiated cells, common myeloid progenitors, multipotent progenitors, and stem cells to replenish their populations by reducing the pressure for their premature differentiation. The experiments that tested the permissiveness of hematopoiesis damaged by ionizing radiation, and rescued by bone marrow transplantation applied shortly after irradiation, demonstrated that hematopoietic tissues remain permissive to transplanted cells after the significant production of blood cells has already resumed, which is particularly valid for the engraftment of stem cells.

## Data Availability Statement

The raw data supporting the conclusions of this article will be made available by the authors, without undue reservation.

## Ethics Statement

The animal study was reviewed and approved by the Laboratory Animal Care and Use Committee of the First Faculty of Medicine, Charles University and the Ministry of Education, Youth and Sports and of the Czech Republic.

## Author Contributions

MB, C-LC, KF, and TH: data collection and analysis, and manuscript writing. KS, PP, and LŠ: data collection and analysis. EN: experimental design, data interpretation, manuscript writing, and final approval of manuscript. All authors contributed to the article and approved the submitted version.

## Conflict of Interest

The authors declare that the research was conducted in the absence of any commercial or financial relationships that could be construed as a potential conflict of interest.

## Publisher’s Note

All claims expressed in this article are solely those of the authors and do not necessarily represent those of their affiliated organizations, or those of the publisher, the editors and the reviewers. Any product that may be evaluated in this article, or claim that may be made by its manufacturer, is not guaranteed or endorsed by the publisher.
