# Supplementary material for: Hematopoiesis Remains Permissive to Bone Marrow Transplantation After Expansion of Progenitors and Resumption of Blood Cell Production
Source: Front Cell Dev Biol. 2021 Aug 3;9:660617. doi: 10.3389/fcell.2021.660617 (PMC8369928; doi:10.3389/fcell.2021.660617)
Supplement: Supplementary file 1 [file Data_Sheet_1.pdf]

Supplementary material

(Báječný et al., Front. Cell Dev. Biol. 9:660617)

Doi: 10.3389/fcell.2021.660617

Supplementary Figure S1

CD71/Sca-1 diagram of LK cells is significantly changed and variable in the hematopoiesis intensively regenerating from endogenous repopulating cells.

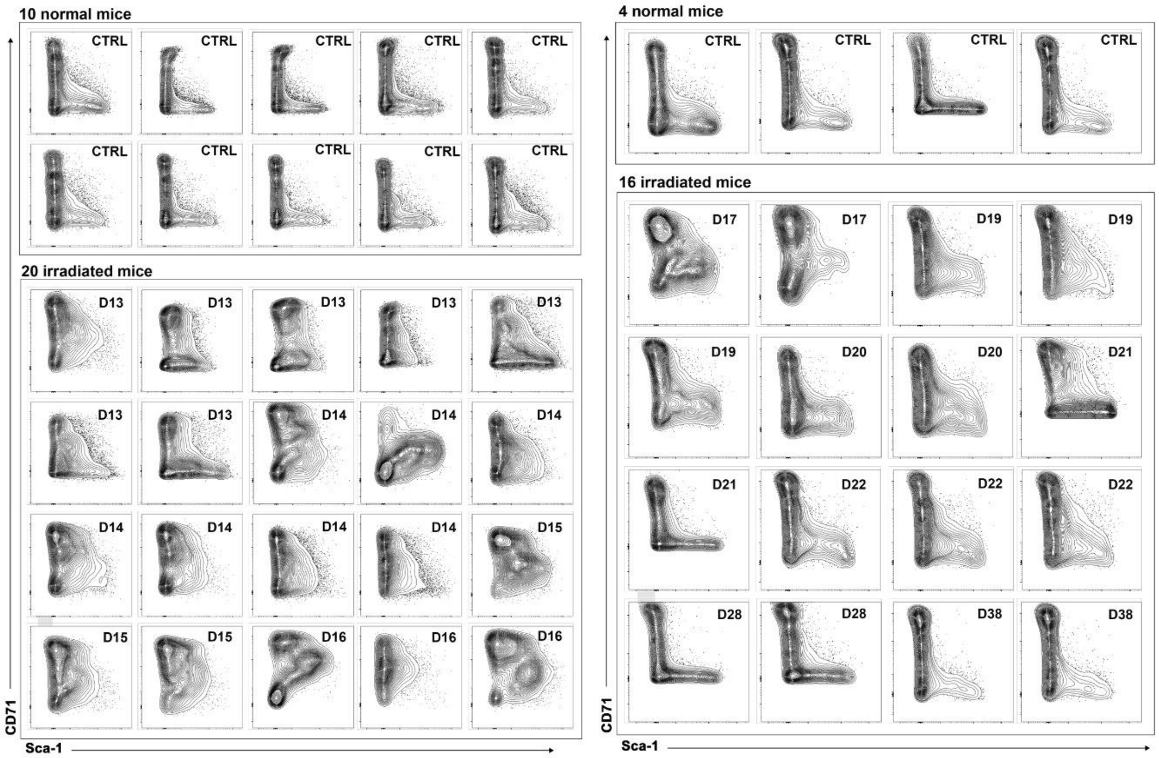

The bone marrow of 14 normal mice (CTRL) and 36 mice irradiated at 6 Gy and analyzed 13-38 days (D13-D38) after irradiation. CD71/Sca-1 plot of Lin-c-Kit+ cells (LK cells) is highly constant in normal bone marrow but is changed and highly variable in intensively regenerating hematopoiesis. Figure 1 in Faltusová et al. (2020) demonstrates the independence of the changes in CD71 and Sca-1 expression from erythropoietin stimulation and exhausted iron body stores.

Faltusová K, Chia-Ling Chen, Tomáš Heizer et al. Altered erythro-myeloid progenitor cells are highly expanded in intensively regenerating hematopoiesis. Front Cell Dev Biol. 8, 98 (2020); doi:10.3389/fcell.2020.00098

Supplementary Figure S2

CD71/Sca-1 diagram of LK cells in the bone marrow of untreated C57Bl/6 mice analyzed by flow cytometry and gene expression.

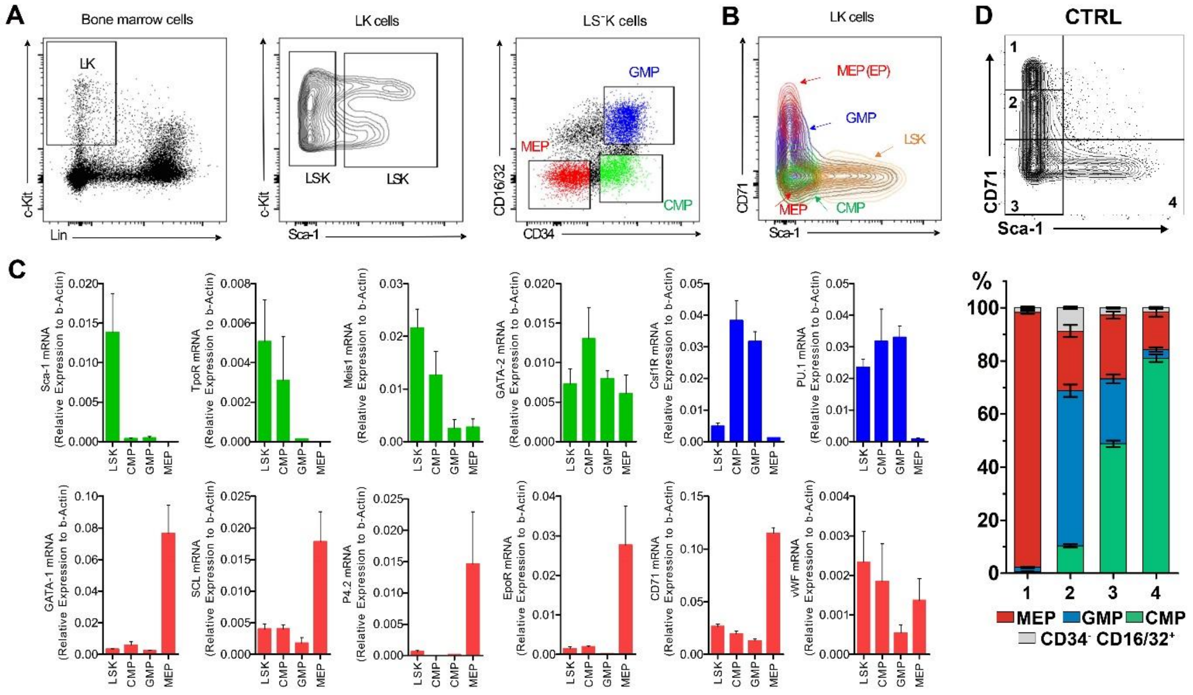

A. RNA was isolated from sorted LSK cells and three subsets of LS⁻K cells (CMP, MEP, and GMP) from normal bone marrow. **B.** We projected these four populations of LK cells into the CD71/Sca-1 diagram. **C.** The mRNAs for twelve genes, those of *Sca-1*, *TpoR*, *Meis-1*, *GATA-2*, *PU-1*, *Csf1-R*, *GATA-1*, *SCL*, *P4.2*, *EpoR*, *CD71*, and *vWF*, were measured by qPCR. LSK cells highly expressed the mRNA for *Sca-1*, *TpoR*, *Meis1*, *GATA-2*, *PU-1*, and *vWF*. CMP expressed the same mRNAs except that for Sca-1 and expressed the mRNA for *Csf1-R*. GMP expressed the mRNAs for *PU-1* and *Csf1-R*. MEP highly expressed the mRNAs for *GATA-1*, *SCL*, *P4.2*, *EpoR*, *CD71*, and *vWF*. The absolute expression level is related to that of the reference housekeeping *ß-Act* gene; results (mean ± SD) are pooled from two independent experiments. **D.** The proportion of CD34/CD16-32 subtypes of LK cells in three groups (1-3) of LS⁻K cells with different expression level of CD71, and in LSK cells (4).

Supplementary Figure 3

CD71/Sca-1 diagram of LK cells in the bone marrow of untreated C57Bl/6 mice analyzed by flow cytometry and gene expression in four types of cells with characteristic expression of CD34, CD16/32, and CD71 markers.

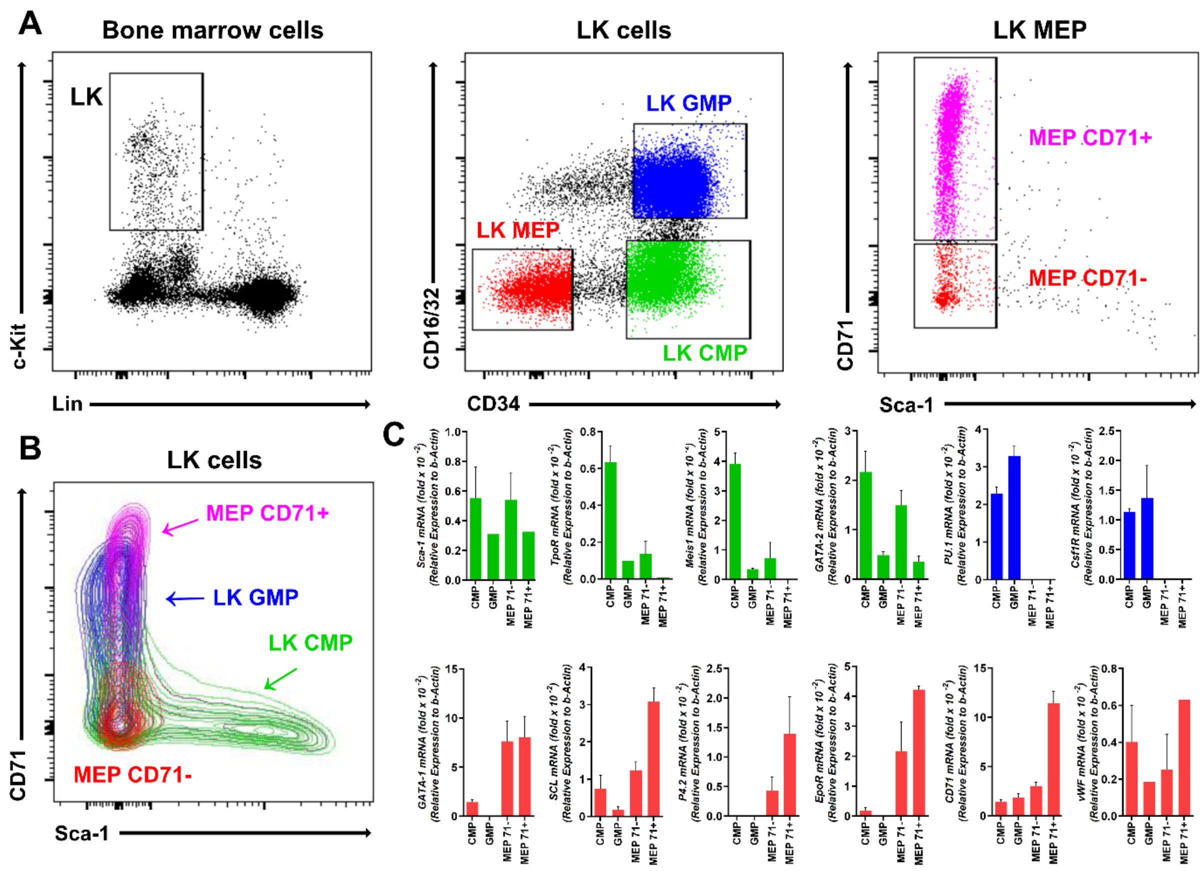

Four subpopulations of LK cells, the CMP, GMP, MEP CD71^{low}, and MEP CD71^{high} cells, were sorted from normal bone marrow. **A.** The cell sorting gate strategy. **B.** The projection of the four sorted cell types into CD71/Sca-1 diagram. **C**. The absolute expression level of twelve genes (as in Supplementary Figure S2) against that of the reference housekeeping gene *ß-Act*; results show mean ± SD. Bone marrow cells were collected from one normal male mouse, and PCR was run in triplicates.

Supplementary Figure S4

LK cells in the chimeric hematopoiesis derived from transplanted and endogenous hematopoiesis reconstituting cells analyzed in the bone marrow and spleen 15 days after irradiation and transplantation.

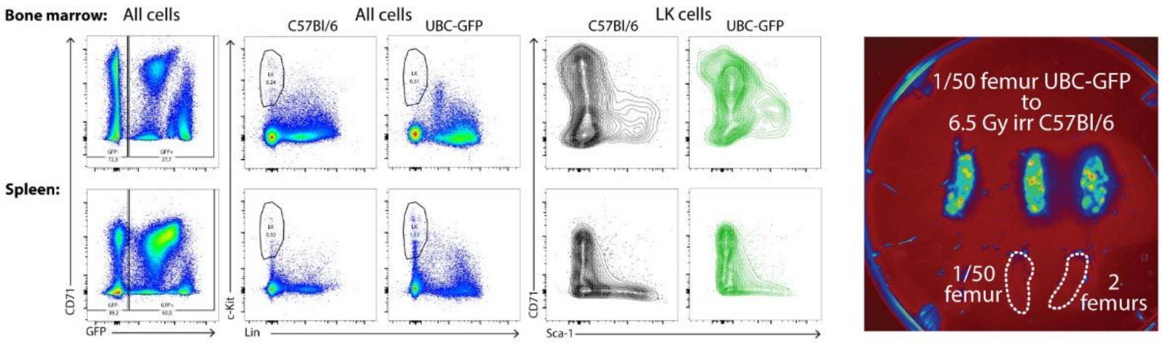

A representative flow cytometry result from three C57Bl/6 female mice irradiated at 6.5 Gy and transplanted with 1/50 equivalent of the femoral bone marrow of UBC-GFP female mice. Two non-irradiated female mice were transplanted with 1/50 and 2 equivalents of the femoral bone marrow of UBC-GFP mice. The bone marrow and spleens were collected after 15 days. The black contours show GFP^{-} LK cells, originating from endogenous hematopoiesis reconstituting cells of C57Bl/6 hosts, while the green contours show GFP^{+} cells originating from transplanted cells. All spleens were examined for GFP fluorescence. Only mice conditioned by irradiation provided significant GFP signal.

Supplementary Figure S5

Transplanted LSK CD48$^-$cells are highly potent for giving rise to populations of LK cells in bone marrow and spleen in contrast to LSK 48$^+$ and LS$^-$K cells.

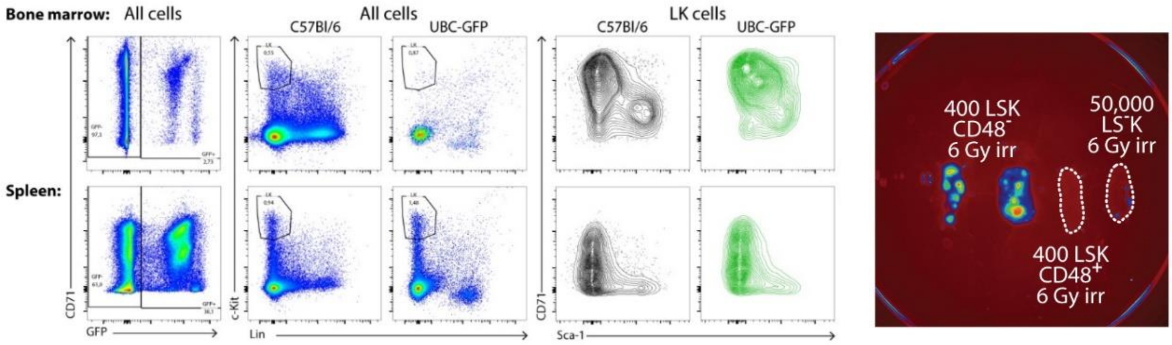

Four C57Bl/6 female mice were irradiated at 6 Gy and transplanted with cells sorted from bone marrow of a UBC-GFP male mouse. 400 LSK CD48$^-$cells (two mice each) or 400 LSK CD48$^+$ or 50,000 LS$^-$K cells (single mice) were transplanted. The peripheral blood, bone marrow, and spleens were collected two weeks after transplantation. A representative result from flow cytometry analysis of LK cells in the bone marrow (tibia) and spleen of one of the two mice transplanted with 400 LSK CD48$^-$cells is shown. The percentage of GFP$^+$ cells in the peripheral blood is in Table 1 in the main body of the article. The black contours show GFP$^-$ LK cells, and the green contours show GFP$^+$ cells. All spleens were examined for GFP fluorescence.

Supplementary Figure S6

The cell gating employed for sorting CD150^{-}or CD150^{+} LSK CD48^{-}cells.

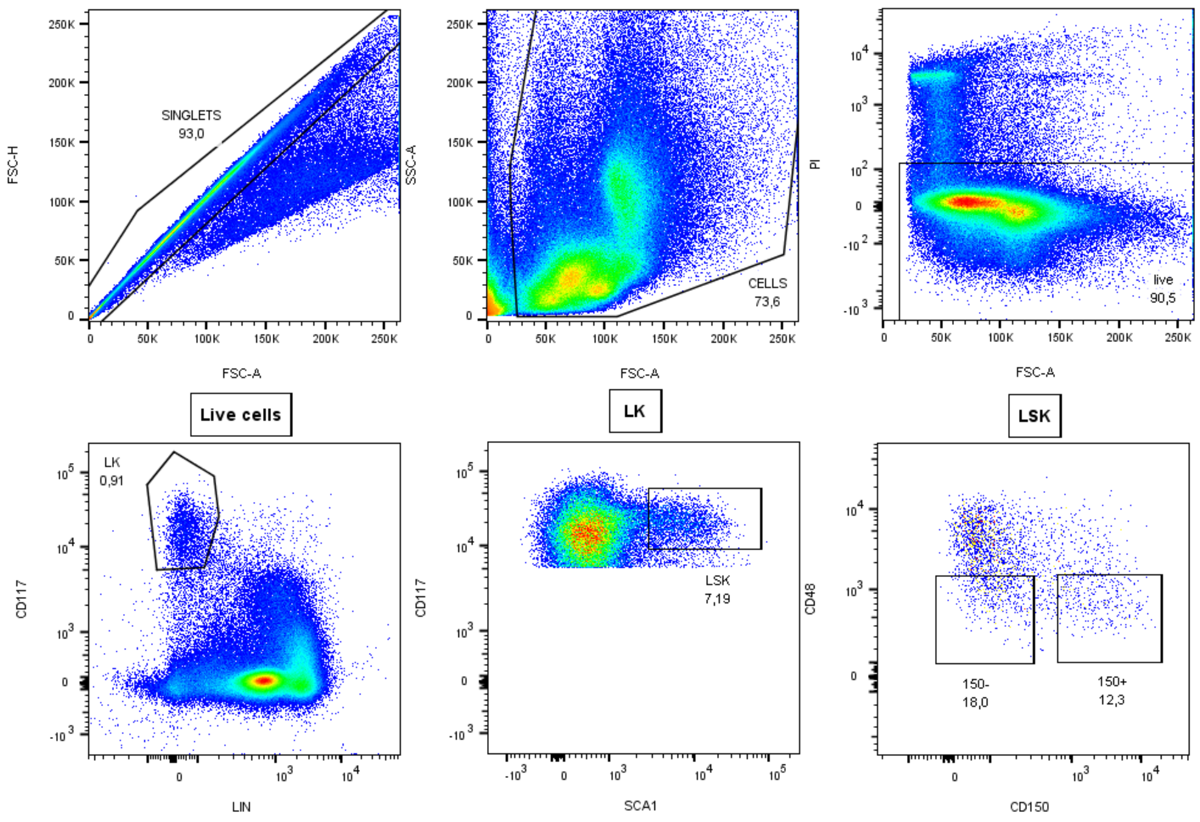

Live (propidium iodide; PI negative) nucleated bone marrow cells were stained for the markers of differentiate lineages (LIN), c-Kit receptor (CD117), Sca-1 antigen (SCA1), CD48, and CD150 markers.

Supplementary Figure S7

LK cells in the chimeric hematopoiesis of ten C57Bl/6 mice irradiated at 6.5 Gy and transplanted with 400 LSK CD48^{-}either CD150^{-}or CD150^{+} cells sorted from the bone marrow of UBC-GFP mice.

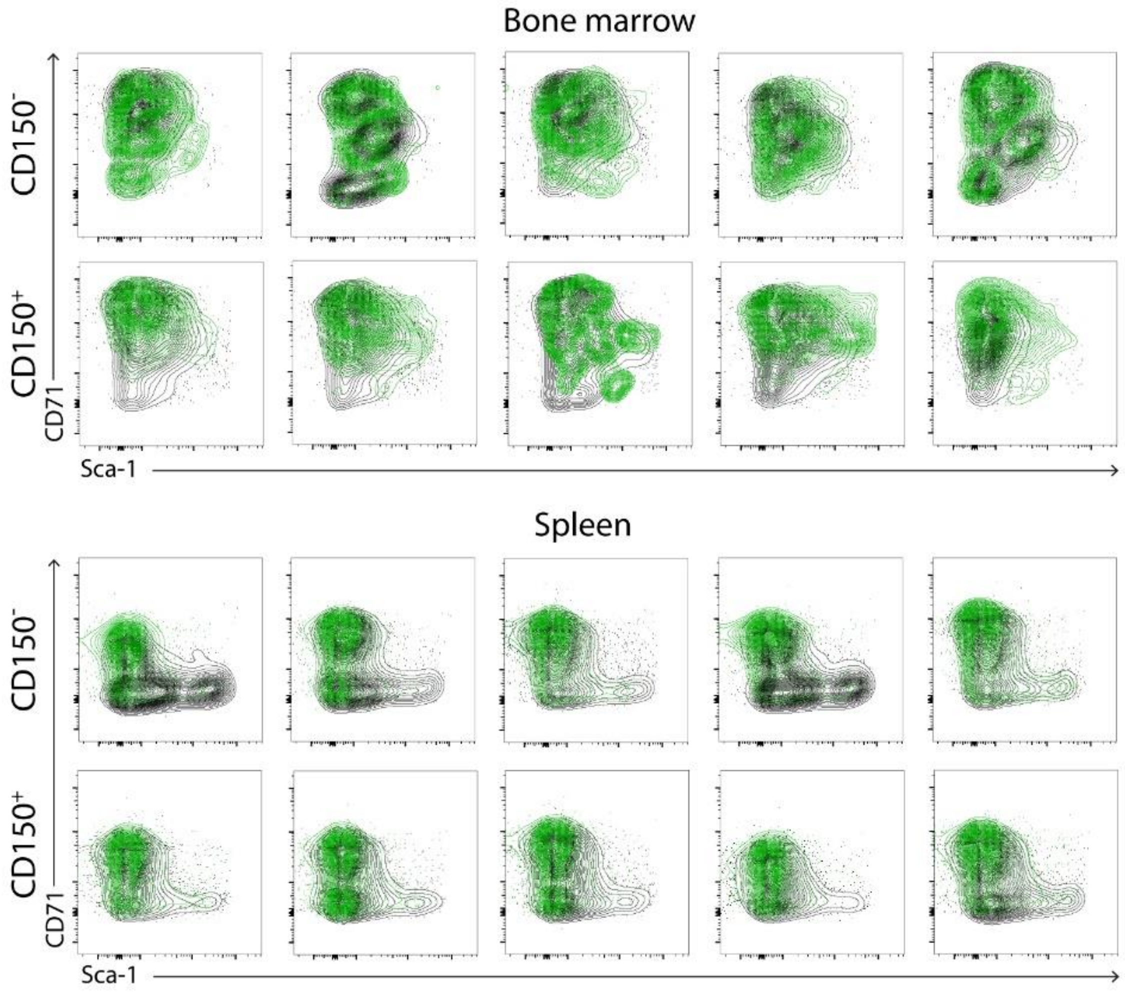

CD71/Sca-1 plot of bone marrow and spleen cells was determined two weeks after transplantation of CD150^{-}and CD150^{+} cells, sorted from bone marrow of untreated UBC-GFP mice, to two groups of five irradiated (6.5 Gy) C57Bl/6 mice. Bone marrow and spleen were examined 14 days after transplantation.

Supplementary Figure S8

Determination of GFP⁺ and GFP⁻ red blood cells (Ter119) and platelets (CD41) in the chimeric UBC-GFP/wild type peripheral blood.

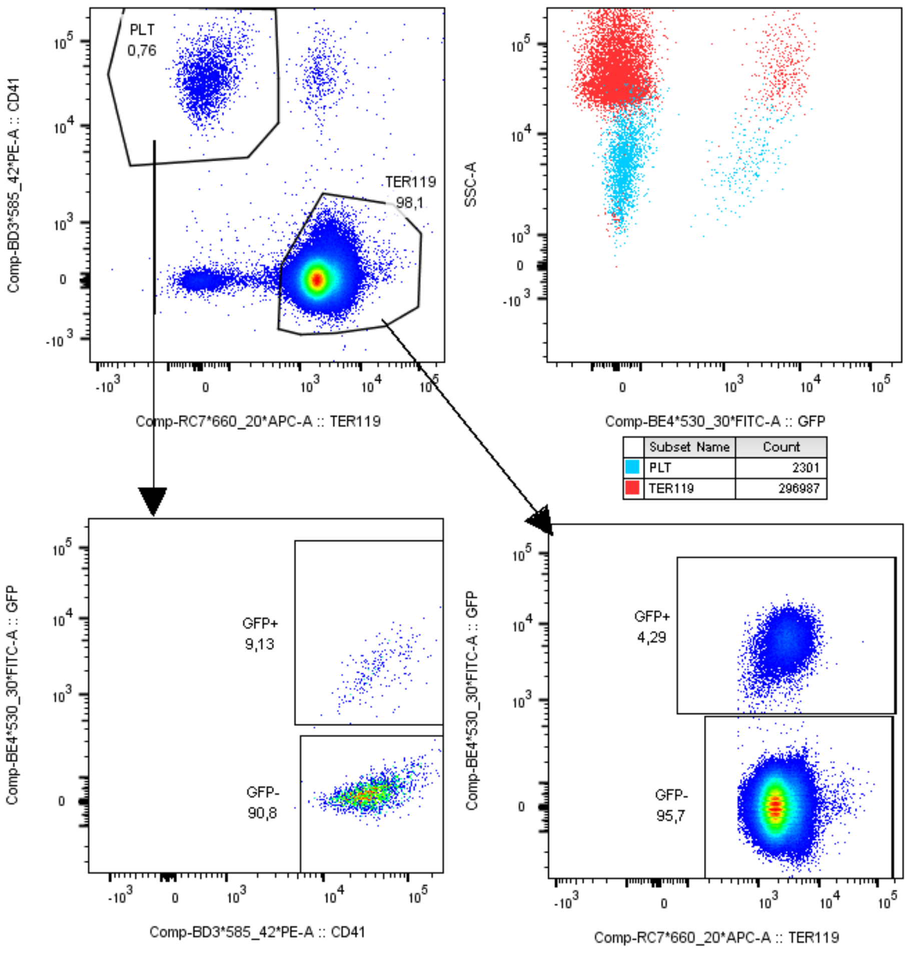

The red blood cells in the peripheral blood were identified as Ter119⁺ cells and platelets as CD41⁺ blood elements. GFP fluorescence allowed discrimination of the blood constituents into those originating from the transplanted cells and the endogenous host´s cells.

Supplementary Figure S9

Graphical presentation of the results also presented as numbers in the main body of the article.

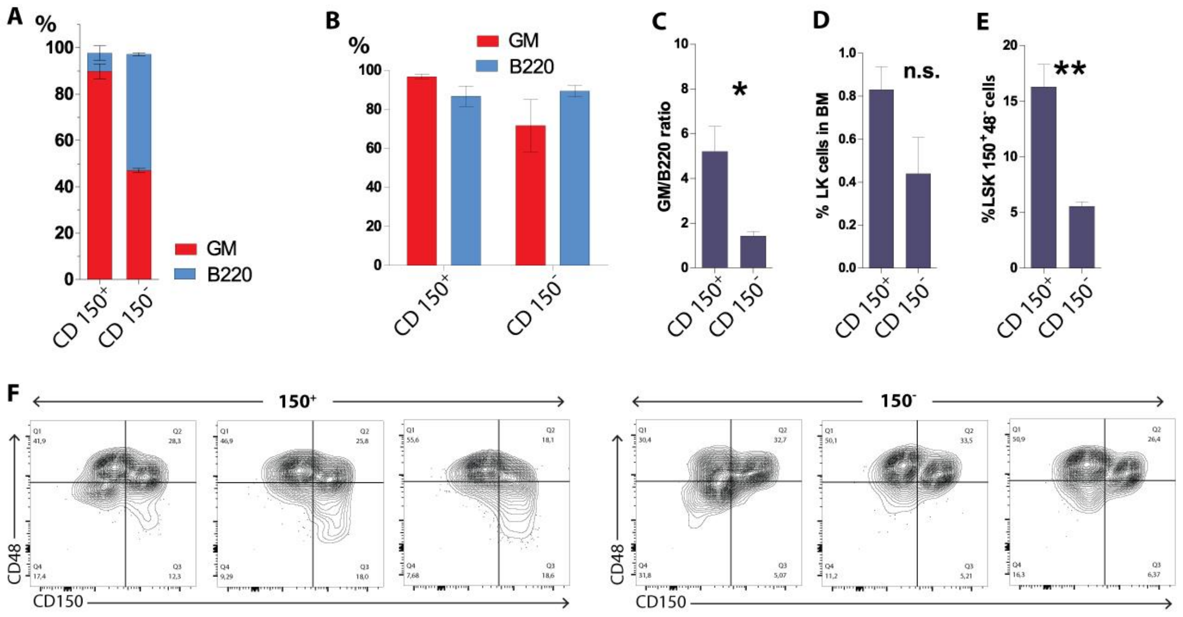

Two groups of three CD45.1 female mice irradiated at 7 Gy received either 3700 LSK CD48^{-} CD150^{+} cells or 5000 LSK CD48^{-} CD150^{-} cells sorted from the bone marrow of two C57Bl/6 female mice (CD45.2). Hematopoiesis of the transplanted mice was examined in the peripheral blood and bone marrow after three weeks.

A. Granulocyte-macrophages (GM) and B-cells (B220) (CD45.2) generated by transplanted LSK CD48^{-}, either CD150^{+} or CD150^{-} cells, in the peripheral blood.

B. The percentage of granulocyte-macrophages (GM) cells and B-cells (B220) of donor origin (CD45.2) in all GM or B220 bone marrow cells.

C. The ratio between GM and B220 cells in all bone marrow cells.

D. The percentage of LK cells in the bone marrow.

E. The percentage of the CD150^{+}CD48^{-} subset of LSK cells.

F. The CD150/CD48 plot of LSK cells in the bone marrow of three mice transplanted with CD150^{+} LSK CD48^{-} cells and three mice transplanted with CD150^{-} LSK CD48^{-}cells.

Supplementary Figure S10

CD16/32+ LSK cells in regenerating bone marrow contain BFU-E and CFU-GEMM next to CFU-G, CFU-M, and CFU-GM.

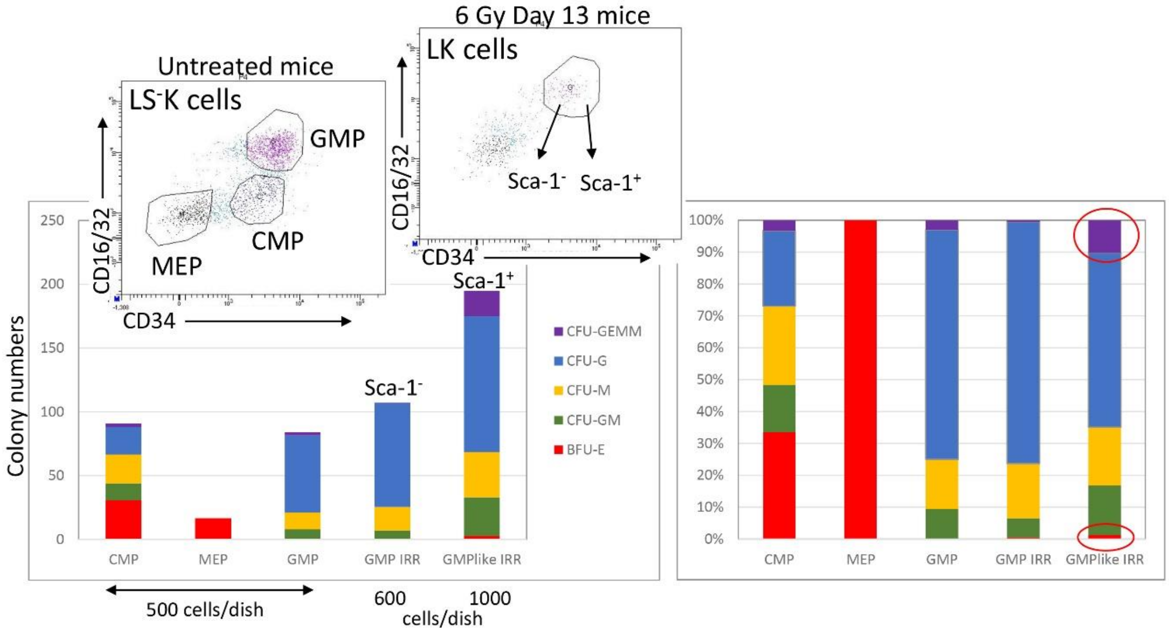

The cells were sorted from pooled bone marrow of two untreated mice and three mice irradiated (IRR) at 6 Gy before 13 days (all mice were CD45.1 males) into methocult M3434 medium (StemCell Technologies, Canada). Cells were cultured in 6 wells plates (Ø 3 cm). Colonies were evaluated after 8 – 10 days.

Supplementary Table S1

Reagents used in immunophenotyping of hematopoietic cells

Antibody	Clone	Producer	Fluorochrome
anti-Lineage cocktail of antibodies CD45R(B220)/CD3/Ly-6G(Ly-6C)/CD11b/Ter-119)		http://www.biolegend.com/	Alexa Fluor® 700
CD117 (c-kit)	2b8	http://www.biolegend.com/	Brilliant Violet 421™
Ly-6A/E (Sca-1)	D7	http://www.biolegend.com/	PerCP
Ly-6A/E (Sca-1)	E13-161.7	http://www.biolegend.com/	PE/Cy7
CD48	HM48-1	http://www.biolegend.com/	PE
CD48	HM48-1	http://www.biolegend.com/	FITC
CD150 (SLAM)	TC15-12F12.2	http://www.biolegend.com/	APC
CD150 (SLAM)	TC15-12F12.2	http://www.biolegend.com/	Brilliant Violet 605™
CD71	RI7217	http://www.biolegend.com/	FITC
CD71	RI7217	http://www.biolegend.com/	PE
CD45.2	104	http://www.biolegend.com/	PE/Cy7
CD41	MWReg30	http://www.biolegend.com/	APC
CD41	MWReg30	http://www.biolegend.com/	PE
CD45.1	A20	http://www.biolegend.com/	APC
CD45R/B220	RA3-6B2	http://www.biolegend.com/	FITC
CD4	53-6,7	http://www.biolegend.com/	PerCP
CD8	GK1.5	http://www.biolegend.com/	PerCP
Ly-6G(Gr-1)	RB6-8C5	http://www.biolegend.com/	Brilliant Violet 421™
CD11b	M1/70	http://www.biolegend.com/	Brilliant Violet 421™

TER-119/Erythroid Cells	TER119	http://www.biolegend.com/	PerCP
CD 127 IL7	93	http://www.biolegend.com/	Brilliant Violet 785™
CD 16/32	A7R34	http://www.biolegend.com/	Brilliant Violet 510™
CD 34	RAM34	http://www.ebioscience.com/	Biotin
CD 135	A2F10	http://www.biolegend.com/	APC
Streptavidin		http://www.biolegend.com/	PE/Cy7

Supplementary Table S2

Primer sequence used for qPCR

HSC/ MPPs/ CMPs		Forward Sequence	Revers Sequence	NCBI accession #
Sca-1	lymphocyte antigen 6 complex	GTTTGCTGATTCTTCTTGTGG	TAACTGCTGCCTCCTGAGTA	NM_001271416.1
c-Kit	KIT proto-oncogene receptor tyrosine kinase	TTACGTGAACACAAAACCAG	CCAAATGGTGACACAGATAC	NM_001122733.1
TpoR	myeloproliferative leukemia virus oncogene	CTGTATGCCTACCGAGGAG	TCTGGTTGAGGGACACATT	NM_001122949.2
Runx1	runt related transcription factor 1	TACTCGGCAGAACTGAGAAA	TGGTAGGTGGCAACTTGTG	NM_001111021.2
HoxB4	homeobox B4	GGAGTTGGAGAAGGAGTTTCAC	AGCGGATCTTGGTGTTG	NM_010459.7
Meis1	Meis homeobox 1	CCTCTTCCCTCTCTTAGCAC	CCTTAACACTTGTATGGCTTG	NM_010789.3
Pbx1	pre B cell leukemia homeobox 1	ATCACAGTCTCCCAGGTATC	GGAGTAGAGGGCGAGTTAG	NM_183355.3
Gata2	GATA binding protein 2	CAACACACCACCCGATAC	TTCTGAGCAGGAGCGAG	NM_008090.5
**GMPs**				
PU.1	spleen focus forming virus (SFFV) proviral integration oncogene	CTTCCCTTATCAAACCTTGTCC	GAACTGGTAGAGGCGAAT	NM_011355.2
Csf1R	colony stimulating factor 1 receptor	GAGCATCTTTGACTGCGT	GCCATTTGGTATCCATCCTT	NM_001037859.2
c/EBPα	CCAAT/enhancer binding protein (C/EBP), alpha	TGTATCTGGTCTCTGTGTCC	GAGTCTCAGTTTGGCAAGAA	NM_007678.3
EVI2B	ecotropic viral integration site 2b	ATCAGCGGTCAACAACT	AGAAGAGGTAGGAGCAGG	NM_001077496.1
c-Myb	myeloblastosis oncogene	CTCCCACACCATTCAAACA	TCCACCTCCTGCTTGATTT	NM_001198914.1
Gfi-1	growth factor independent 1	ACATCTGCTCATTCACTCG	CCTGTGTGCTTTCTGCTA	NM_010278.2
**Lyms**				
Flt3	FMS-like tyrosine kinase 3	AGTTCAGTTACACCCGCC	GCAAAGCAAAGGAGGTCT	NM_010229.2
Notch1	notch 1	TGTGGCTTCCTTCTACTGCG	TTTGCCGTTGACAGGGTT	NM_008714.3
Irf8	interferon regulatory factor 8	TTACAATCAGGAGGTGGATGC	CGGTCAGTCACTTCTTCAAAAT	NM_001301811.1
**MEPs**				
Gata1	GATA binding protein 1	AGTGTGTGAACTGTGGAGCA	GAGTGTTGTAGTGGTCGTTT	NM_008089.2
EpoR	erythropoietin receptor	GAGCACCTATGACCACCCA	CACTCCAGAATCCGCTGAA	NM_010149.3
P4.2	erythrocyte membrane protein band 4.2	CAAACGGGAGAGCAACC	ACCTGAAGCAAGAGTGAG	NM_013513.3
Klf1	Kruppel-like factor 1 (erythroid)	GGCGAACTTTGGCACCTAA	ATAAGGCTTCTCTCCCGT	NM_010635.2
CD71	transferrin receptor	GGCTACCTGGGCTATTGTAA	GGTGTCAGCAAACTCTATG	NM_011638.4
Scl	T cell acute lymphocytic leukemia 1	TCAATGACCAGGAGGAGGAA	GGGAAGTGTGCTTGGGTGTT	NM_011527.3
Fog1	zinc finger protein, multitype 1	ATCCCCTGAGAGAGAAGAAC	CCAGAAGGCACCACTTT	NM_009569.4
NF-E2	nuclear factor, erythroid derived 2	TGGAGAGATGGAACTGACT	ATAAGATGGTGGGGGAAGG	NM_001302338.1
vWF	Von Willebrand factor	CAGGTGTCCGTGATACAGT	GCTCCGTGGATTTGTGAAG	NM_011708.4

Fli1	Friend leukemia integration 1	GGGGAGTTCAAAATGACGG	GGATGTCTCTGTTGGATGTG	NM_008026.5
Eto2	CBFA2/RUNX1 translocation partner 3	CAGAAGAATGGAAGCACCT	GTTCCATCTTGGCACGC	NM_009824.2
**Housekeeping genes**				
Actb	musculus actin, beta	AGACTTCGAGCAGGAGAT	ATGCCACAGGATTCCATAC	NM_007393.5
